# Consultation-based interventions to optimize medication adherence in primary care: a systematic review

**DOI:** 10.1093/fampra/cmag007

**Published:** 2026-03-03

**Authors:** Caitriona Callan, Jadine Scragg, Richard Stevens, Laura Heath, Isabella De Vere Hunt, Anna Seeley, Alexandra Caulfield, Paul Aveyard

**Affiliations:** Nuffield Department of Primary Care Health Sciences, University of Oxford, Oxford, United Kingdom; Nuffield Department of Primary Care Health Sciences, University of Oxford, Oxford, United Kingdom; Nuffield Department of Primary Care Health Sciences, University of Oxford, Oxford, United Kingdom; Nuffield Department of Primary Care Health Sciences, University of Oxford, Oxford, United Kingdom; Nuffield Department of Primary Care Health Sciences, University of Oxford, Oxford, United Kingdom; Nuffield Department of Primary Care Health Sciences, University of Oxford, Oxford, United Kingdom; Nuffield Department of Primary Care Health Sciences, University of Oxford, Oxford, United Kingdom; Nuffield Department of Primary Care Health Sciences, University of Oxford, Oxford, United Kingdom

**Keywords:** medication adherence, behavioral medicine, noncommunicable diseases, primary health care, systematic review, meta-analysis

## Abstract

**Objective:**

To synthesize evidence on the effectiveness of consultation-based interventions on adherence to primary or secondary preventative medications and clinical outcomes. We focused on consultation-based interventions suitable for primary care settings, without needing specific technologies, and with reasonable time requirements of clinicians.

**Methods:**

A database search was undertaken from 2015 onwards, supplemented by previous systematic reviews and citation-searching. Randomized trials targeting adults prescribed long-term medication for cardiovascular prevention, type 2 diabetes mellitus (T2DM), chronic respiratory disease, or osteoporosis were included. Interventions had to meet *a priori* eligibility criteria for implementation feasibility in primary care. Two reviewers screened, extracted data, and assessed risk of bias using the Cochrane RoB2 tool. Adherence and clinical outcomes were assessed, with meta-analyses conducted using inverse variance heterogeneity methods and sensitivity analyses to explore heterogeneity.

**Results:**

41 studies (*n* = 26 114) were included. Meta-analysis showed significant adherence improvements for T2DM [standardized mean difference (SMD) 0.60, 95% confidence interval (CI) 0.10 to 1.11] and chronic respiratory disease (SMD 0.22, 95% CI 0.07 to 0.38), but effects were not robust to sensitivity analyses. No significant adherence effects were observed for cardiovascular prevention nor osteoporosis. Interventions did not significantly improve clinical outcomes including systolic blood pressure, low-density lipoprotein, HbA1c (after sensitivity analyses), respiratory symptoms, or hospitalization. High heterogeneity and study-level risk of bias limited certainty.

**Conclusion:**

Consultation-based interventions may modestly improve medication adherence in T2DM and chronic respiratory disease, but there is no robust evidence of clinical benefit, nor evidence of effectiveness in other conditions. Intervention feasibility is an important consideration for guiding future research and translating it into practice.

Key messages

- Improving adherence to preventative medication is a health system priority.
- This systematic review and meta-analysis analysed consultation-based interventions.
- There was limited and low-certainty evidence interventions improved adherence.
- There was no robust evidence of clinical benefit from interventions.
- Trials should consider interventions' demands of staff capability and time.

## Background

Many health systems are trying to reorientate to prevention, aiming to improve outcomes for patients and reduce healthcare costs. An important means to achieve this is through preventative medication. While the majority of regular medications prescribed in primary care is for primary or secondary prevention [1], medication adherence has been estimated in systematic reviews to be in the range of 30–50% [2, 3]. Non-adherence increases the risk of poor health outcomes: a consistent risk factor for high mortality and morbidity accounting for potential confounders in several meta-analyses [4-7]. Nine percent of all cardiovascular events may be attributable to poor adherence [4]. Improving adherence to long-term preventative medications is, therefore, a key health system priority to improve individual and population health outcomes [8, 9].

Initiating and supervising preventative medication mainly takes place in primary care. In practice, clinicians approach this like any other prescription, but for the patient, the rationale of medication use, particularly for symptomless conditions or to prevent a disease they have not had, may be unclear, and the implications of lifelong medication may loom large. The Perceptions and Practicalities Approach (PAPA) suggests that patients' intentions to adhere are based on the balance the necessity of taking medication against the concerns they may have about doing so, and this intention is moderated by the practicalities of adherence [10, 11]. Interventions to improve adherence may relate to one or more of these constructs.

Given the time constraints of practice, interventions that aim to increase adherence to medication need to be incorporated into the medication consultation and thus need to be feasible and brief [12]. Several health systems internationally have therefore sought to improve medication adherence through pragmatic interventions delivered through consultations when initiating or reviewing long-term preventative medications [13-15]. The NHS New Medicines Service (NMS) utilizes community pharmacists to help patients manage new medications for long-term conditions, with evaluation at 6 months finding a modest improvement in medication adherence. Although this improvement did not meet prespecified significance levels, economic modelling suggested the service could still improve patient outcomes compared with usual care [15].

### Objective

In this systematic review, we assessed the effectiveness of brief consultation-based interventions in primary care in improving adherence to, and relevant clinical outcomes of, long-term medication for primary or secondary prevention. We focussed on consultation-based interventions which could be used in any primary care setting without needing specific technologies, which were also reasonable in their time requirement of clinicians.

## Methods

A full protocol was prospectively published on the International Prospective Register of Systematic Reviews [16], and we followed the PRISMA (Preferred Reporting Items for Systematic reviews and Meta-Analyses) guidelines [17].

### Eligibility criteria

We included randomized trials (RCT) and cluster randomized trials (cRCT) conducted in primary care or hospital outpatient settings. The population considered for inclusion were adult patients (18 years of age or over) being prescribed medication for primary or secondary prevention of cardiovascular disease, type 2 diabetes mellitus (T2DM), chronic respiratory disease [asthma or chronic obstructive pulmonary disease (COPD)], or osteoporosis and which assessed a relevant clinical outcome.

Trials were excluded if they focussed on haemodialysis, transplant, or paediatric populations; symptomatic treatments, e.g. treatment for COPD exacerbation; people in institutional settings, lacking mental capacity around medications; or thought to be in the last year of life.

Interventions included were those aimed at increasing medication adherence, which met *a priori* inclusion criteria for feasibility of implementation in routine primary care. These criteria, drawing from characteristics of existing interventions, such as the NHS NMS, deployed at national scale aiming to improve adherence [13-15, 18], were developed by the authorship team (which included five UK general practitioners) based on three factors described in Table 1.

**Table 1:** Summary of intervention and outcome inclusion criteria.

Intervention inclusion criteria	Description	Illustrative examples of included interventions
1. Deliverable in a primary care consultation	Change in care delivered as part of (face to face or remotely delivered; individual or group) patient consultation.	Structured discussion of personalised risk score [19, 20] Use of decision aid in consultation on choice of diabetes medication [21-23] Clinician communication skills or shared decision-making training, with intention of changing how consultations undertaken [24, 25]
2. Skills present in primary care team to deliver intervention	Intervention delivered by staff in roles present in primary care multi-disciplinary team, based on Royal College of General Practitioners definition [26]	Discussion of patient-specific barriers to adherence by GPs [27] or pharmacists [28] Medication counselling for new medications for secondary prevention by pharmacists [29, 30] or nurses [31]
3. Feasible in time constraints	Total number of patients contacts 2 or fewer Total interventionist time required per patient 1 hour or less If clinician training with no direct patient intervention, training of less than 8 h	Adherence support session (20 min), and shorter reinforcement session (10 min) [32] One goal setting consultation with one further goal checking consultation 3 months later [33] Two 1-h workshops for clinicians [34]
**Primary outcome inclusion criteria**		
1. Behavioural adherence measure	Objective or subjective At 3 months or more follow-up	Proportion of days covered by filled prescriptions Score on Medication Adherence Report Scale
2. Clinical outcome	Relevant to the target disease of the intervention At 3 months or more follow-up	SBP HbA1c Asthma Control Test [27] COPD Assessment Test [35]

Interventions in which the core change in care was not delivered through consultation, such as financial incentives, automated reminders, self-guided online education, or peer or relative support, were excluded as being less generalizable to implement by any primary care team. Further details of inclusion criteria for interventions and primary outcome measures can be found in Supplementary Tables S1–S3.

### Data sources and searches

Studies included in two previous large systematic reviews of medication adherence interventions, published in 2014 and 2016 [36, 37] were included in abstract screening, and searches of medical databases for further relevant titles and abstracts were conducted from 2015 onwards. A search strategy was developed with a research librarian, using search terms for randomized trials, medication adherence, the disease groups of interest, and relevant drug classes. There was no language restrictions, with all languages translatable by machine software included for screening. The complete database search strategy is included in Supplementary File S1.

### Screening

After deduplication, two reviewers independently screened all title and abstracts and then full-text records against prespecified eligibility criteria using a decision flowchart. Covidence screening software was used for deduplication, and for title, abstract, and full-text screening [38]. Disagreements were resolved by consensus, and where information relating to eligibility criteria was unclear, we contacted authors.

Data extraction was undertaken by two reviewers independently, using a piloted Microsoft Excel spreadsheet. Information extracted and tabulated included baseline characteristics, study design, intervention characteristics (number of contacts required, whether targeting perceptual factors, practicalities, or both according to the PAPA [10]) and adherence and clinical outcome measures. Secondary outcomes of patient and healthcare practitioner acceptability, and health equity impact, were also extracted where reported. Quality assessment data were extracted, with risk of bias independently assessed by two authors using the Cochrane Risk of Bias 2 Tool [39] for the appropriate study design. Disagreements were resolved by consensus.

### Data synthesis and analysis

For outcomes in which the same effect measures were reported across studies [blood pressure, low-density lipoprotein (LDL) cholesterol, and HbA1c], change scores (adjusted for clustering where appropriate, and adjusted for other factors if reported) were meta-analysed and presented as mean difference.

For outcomes in which different effect measures were reported across studies (medication adherence, respiratory disease symptom scores), standardized mean difference (SMD) was calculated at endpoint. For dichotomous effect measures of these outcomes, odds ratio was calculated from reported endpoint data and converted to SMD by the Chinn formula [40]. Methods of imputation and conversion to SMD used are reported in Supplementary File S1.

All effect estimates were reported with a 95% confidence interval (CI) and 95% prediction intervals (PIs) (an interval estimate that expresses the range within which the effect of a new study, similar to those included in the meta-analysis, is expected to lie). As per our registered protocol, the primary analysis used inverse variance heterogeneity (IV-Het) meta-analysis, with Cochran's *Q* and *I*^2^ statistic used to quantify heterogeneity and grouped studies by disease subgroup. Sensitivity analyses were carried out using a random effects method and a random effects analysis with Hartung–Knapp/Sidik–Jonkman (HKSJ) CIs.

Prespecified analysis examined possible causes of heterogeneity by excluding studies at high risk of bias, excluding studies in which participants did not have poor adherence or disease control at baseline. We also undertook subgroup analysis to examine the effectiveness of different types of interventions and number of patient contacts involved in the intervention.

For analyses with at least 10 studies, funnel plots were examined for asymmetry to test for small study effects or reporting bias.

The GRADE (Grading of Recommendations Assessment, Development and Evaluation) approach was used to assess certainty of evidence [41].

## Results

**Figure 1:**
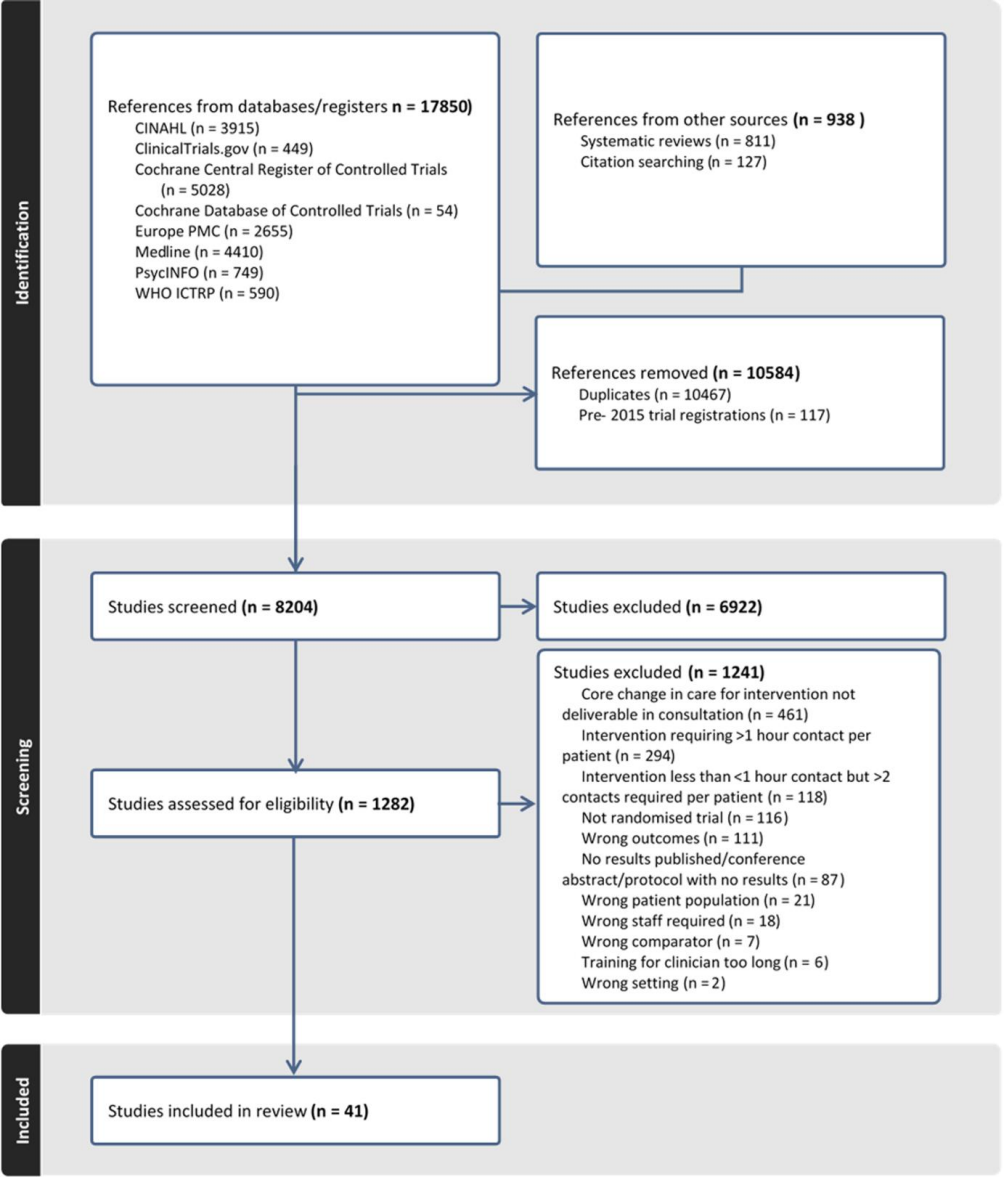
Evidence search and selection.

Figure 1 shows the flow through the study. 17 850 references were identified by search, with a further 938 identified via the previously published systematic reviews and by handsearching included studies. After screening, 1282 full texts were assessed for eligibility and 41 studies were included.

### Study characteristics

Of the 41 included studies, 24 were RCTs including some 7682 participants and 17 were cRCTs including 18 432 participants.

Twenty studies focussed on cardiovascular disease (11 primary, 9 secondary), 8 on T2DM, 8 on chronic respiratory disease (5 asthma, 2 COPD. 1 either or mixed asthma/COPD), 1 on osteoporosis, and 4 which included participants with one or more of primary/secondary cardiovascular prevention or T2DM. The average age of patients was 58.2 (SD 19.9), with 50.5% female. Of the small number of studies (14) that reported ethnicity, 53.8% participants were White. The most common countries were the USA (13), UK (4), Australia (4), Germany (3), and India (2).

Most interventions included were assessed as targeting both perceptual and practicalities according to the PAPA (24 studies), with a smaller number targeting perceptual factors only (15 studies) or not reporting sufficient information to determine the focus of the intervention (2). No studies targeted solely practicalities. There was a wide range of interventions trialled in the included studies. Table 1 provides an overview of the different consultation-based interventions judged to be feasible within a primary care setting. Supplementary Table S4 contains details of all included interventions.

### Risk of bias

Risk of bias for the outcome of medication adherence was judged to be low for 5 studies, of some concern for 6 studies, and high for 30 studies (Supplementary File S1). The most frequent reason for studies being judged at high risk of bias was related to participant loss to follow-up, as missing data due to loss to follow-up was unlikely to be independent from the outcome of medication adherence [39].

### Medication adherence outcome

**Figure 2:**
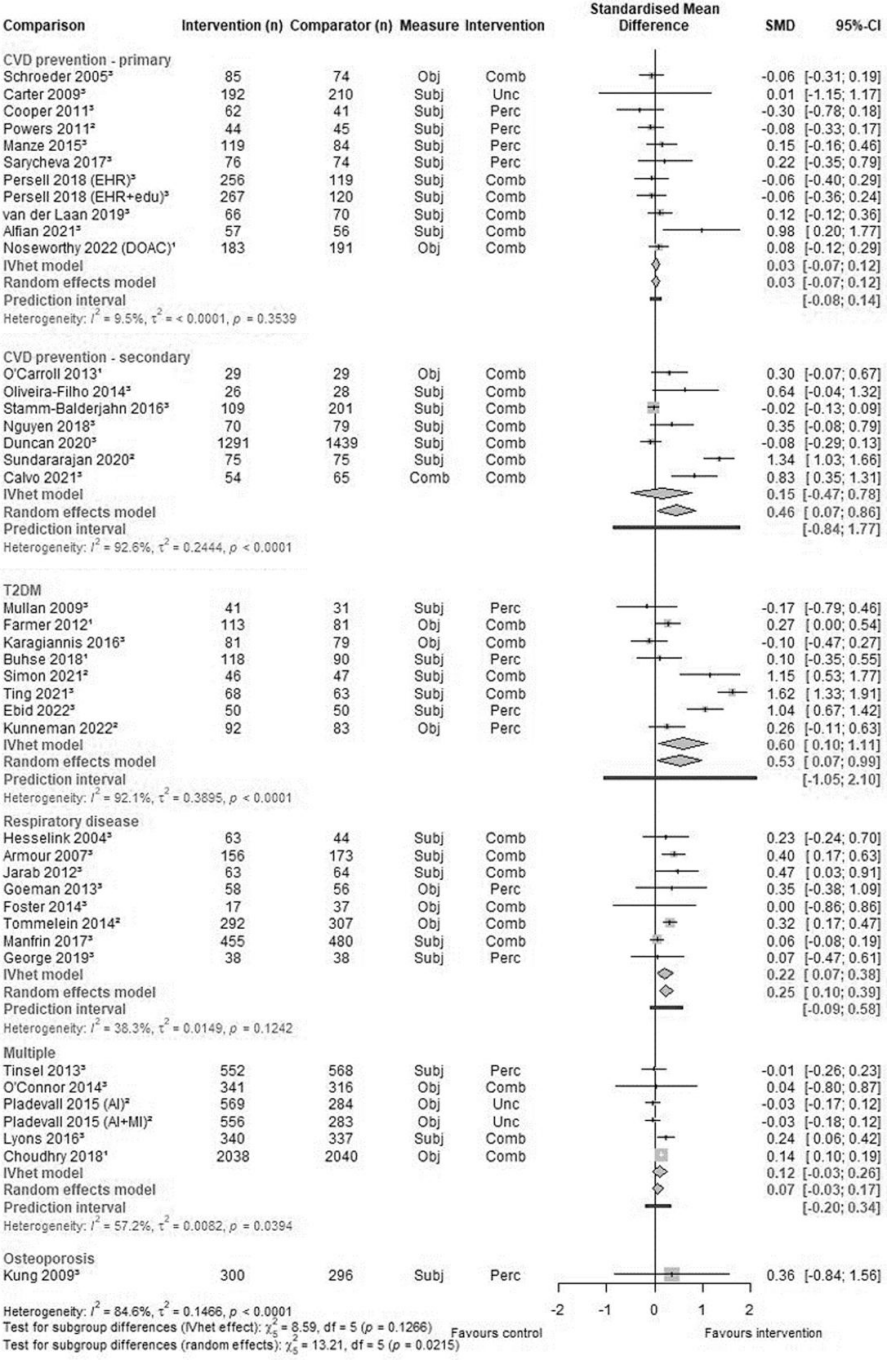
Primary meta-analyses: standardised mean difference in medication adherence at endpoint. Objective measures of adherence (Obj, *n* = 13) included: medication electronic monitoring (5), electronic prescribing data (8). Subjective measures of adherence (Subj, *n* = 25) included: Morisky Medication Adherence Scale (MMAS, *n* = 9), Medication Adherence Report Scale (6), Hill Bone scale (1), Diagnostic Adherence to Medications scale (1), Beliefs about Medicines questionnaire (1), Self-Efficacy for Appropriate Medication Use (1), or medication recall (6). One study used a combined measure of adherence (Comb) including MMAS, prescribing data, Haynes–Sackett score, and attendance at clinic visits. Interventions classified using Perceptions and Practicalities Approach as Perceptual (Perc), Combined (Comb), or Unclear (Unc).

A total of 43 comparisons from 41 studies reported medication adherence outcome, with 41 comparisons from 39 studies included in medication adherence outcome meta-analysis (Fig. 2).

Due to substantial heterogeneity (*I*^2^ = 84.6%), we did not calculate an overall pooled effect estimate for the primary outcome meta-analysis. Observed statistical heterogeneity was also above 50% in all subgroups except primary CVD prevention and chronic respiratory disease. Certainty of evidence for medication adherence in all subgroups was judged to be low or very low (Supplementary Table S5).

Twelve comparisons from 11 studies reported intervention effects on adherence to medications for primary CVD prevention. One study could not be included in meta-analysis, which found no significant intervention effect on medication adherence [24]. Meta-analysis of the remaining 11 comparisons from 10 studies found no significant effect (SMD 0.03, 95% CI −0.07 to 0.12).

Nine studies reported intervention effects on adherence to medications for secondary CVD prevention. Two studies were not included in the meta-analysis, one finding no difference between intervention and control groups [19] and one finding modestly but significantly higher adherence scores in the intervention group compared with control [42]. The meta-analysis of the remaining seven studies found no significant effect, but statistical heterogeneity was high (SMD 0.15, 95% CI −0.47 to 0.78).

Eight studies reported effects of interventions on adherence to medications for T2DM, with meta-analysis finding statistically significantly higher medication adherence in favour of intervention, although with high statistical heterogeneity (SMD 0.60, 95% CI 0.10 to 1.11, 95% PI −1.05 to 2.10). This SMD would be approximately equivalent to intervention resulting in patients taking medication for 13 more days in every 100 (calculated using pooled variance of 22% from standard deviations for proportion of days covered in included studies [21, 22]). Similar results were found in sensitivity analyses using random effects methods (Fig. 2 and Supplementary Figure S1). However, sensitivity analyses excluding studies at high risk of bias and limiting meta-analysis to studies in which participants had poor medication adherence at baseline found no significant effect on adherence (SMD 0.32, 95% CI −0.07 to 0.71 and SMD 0.22, 95% −0.21 to 0.65, respectively). Interventions in this subgroup (detailed in Supplementary Table S4) included use of decision aids, structured patient education, and healthcare provider training in shared decision-making.

Eight studies reported effects of interventions on adherence to medications for chronic respiratory disease, with meta-analysis finding a small but statistically significant difference in medication adherence in favour of intervention (SMD 0.22, 95% CI 0.07 to 0.38, 95% PI −0.09 to 0.58). This SMD would be approximately equivalent to intervention resulting in a 7.1% absolute increase in adherence to an inhaler (calculated using pooled variance of 32% from standard deviations for adherence measured by smart inhaler reported in included studies [27, 43]). Sensitivity analyses using random effects methods found similar results (Fig. 2 and Supplementary Figure S1), while only one respiratory disease study was not rated at high risk of bias (with SMD 0.32, 95% 0.17–0.47), and none focussed on participants with poor medication adherence at baseline. Interventions in this subgroup (detailed in Supplementary Table S4) included goal setting and review, shared decision-making consultations, and structured patient education.

Six comparisons from five studies reported effects of interventions on adherence to medications for studies including patients with either or more than one of T2DM/primary/secondary CVD prevention. Meta-analysis found no significant effect; statistical heterogeneity was high (SMD 0.12, 95% CI −0.03 to 0.26).

Only one study [44] was included reporting effect of interventions on adherence to medications for osteoporosis, finding no significant difference in adherence (SMD 0.36, 95% CI −0.84 to 1.56).

### Funnel plot and subgroup analysis

A funnel plot of all studies showed asymmetry (Supplementary Figure S6), in the form of statistical outliers among studies of chronic respiratory disease and T2DM.

Subgroup analysis of studies which only included participants with poor adherence at baseline, and thus greater potential for improvement in adherence, did not show any significant change in adherence because of interventions (Supplementary Figure S3). Subgroup analyses by type of intervention, and by number of contacts involved in the intervention, showed no significant effect of perceptual or combined interventions, nor of interventions with 0–2 numbers of patient contacts, and no statistically significant subgroup differences (Supplementary Figures S4 and S5).

### Clinical outcomes

Clinical outcomes are summarized in Table 2, with corresponding figures presented in Supplementary File S1. HbA1c in patients with T2DM were the only subgroup where there was evidence of an effect of adherence interventions in primary meta-analysis (Supplementary Figure S12). However, this finding was not robust to sensitivity analyses excluding studies at high risk of bias nor including only studies with poor control at baseline (Supplementary Figures S14 and S15). Funnel plot analysis for this outcome showed notable asymmetry (Supplementary Figure S16), and *post-hoc* analysis excluding one outlier study based on this showed a null result (Supplementary Figure S17).

**Table 2:** Summary of clinical outcomes.

Outcome Disease group	Number of comparisons (participants)	Mean difference and 95% confidence intervals in intervention group compared with control IVHet	Mean difference and 95% confidence intervals in intervention group compared with control Random effects (HKSJ)	Mean difference and 95% confidence intervals in intervention group compared with control excluding studies at high risk of bias	Mean difference and 95% confidence intervals in intervention group compared with control including only studies with poor control at baseline
**SBP**					
CVD prevention—primary	9 (2027)	−0.85 mmHg −3.03; 1.33 I² = 9.4%	−0.9 mmHg −3.45; 1.65	1 (89) 1.9 mmHg −7.35; 11.15	7 (1449) −0.71 mmHg −3.05; 1.63 I² = 28.4%
CVD prevention—secondary	2 (151)	−0.19 mm Hg −9.88; 9.51 I² = 62.5%	1.3 mmHg −58.61; 61.22	2 (151) −0.19 mmHg −9.88; 9.51	0
T2DM	1 (93)	−4.40 mmHg −14.88; 6.08	−4.40 mmHg −14.88; 6.08	−4.40 mmHg −14.88; 6.08	0
Either or multiple of CVD primary/secondary prevention, and T2DM	3 (2536)	0.53 mmHg −1.50; 2.57 I² = 41.3%	0.46 mmHg −4.05; 4.97	1 (1015) 2.1 mmHg −1.47; 5.67	2 (1746) −0.22 mmHg −3.97; 3.53 I² = 62.5%
**LDL**					
CVD prevention—secondary	2 (291)	0.81 mg/dl −5.49; 7.10 I² = 0%	0.81 mg/dl −12.86; 14.48	1 (150) 2.24 mg/dl −8.24; 12.72	0
T2DM	2 (193)	−7.32 mg/dl −34.99; 20.34 I² = 84.2%	−15.91 mg/dl −158.28; 126.46	1 (93) −28.84 mg/dl −46.04; −11.64	1 (93) −28.84 mg/dl −46.04; −11.64
Either or multiple of CVD primary/secondary prevention, and T2DM	4 (5201)	0.39 mg/dl −2.02; 2.80 I² = 0%	0.39 mg/dl −1.81; 2.60	3 (4662) 0.05 mg/dl −46.04; −11.64	4 (5201) 0.39 mg/dl −2.02; 2.80 I² = 0%
**HbA1c**					
T2DM	7 (1052)	**−0.86% glycated haemoglobin** **−1.69; −0.02** I² = 95.2%	−0.53% glycated haemoglobin −1.19; 0.13	3 (559) −0.09% glycated haemoglobin −0.64; 0.47 I² = 67.6%	6 (952) −0.15% glycated haemoglobin −0.78; 0.47 I² = 79.3%
Either or multiple of CVD primary/secondary prevention, and T2DM	4 (3149)	−0.05% glycated haemoglobin −0.24; 0.13 I² = 44.6%	−0.06% glycated haemoglobin −0.33; 0.22	3 (2180) −0.15% glycated haemoglobin −0.30; 0.00 I² = 0%	4 (3149) −0.05% glycated haemoglobin −0.24; 0.13 I² = 44.6%
**Hospitalization**					
Primary or secondary CVD prevention	6 (1394)	OR 1.03 0.6; 1.78 I² = 0%	OR 1.03 0.57; 1.86	1 (915) OR 1.16 0.33; 4.14	N/A
**Respiratory symptoms**					
Chronic respiratory disease	8 (2445)	Standardised mean difference −0.01 −0.1; 0.08 I² = 0%	Standardised mean difference −0.01 −0.07; 0.05	1 (692) Standardised mean difference −0.08 −0.82; 0.66	3 (487) Standardised mean difference −0.12 −0.53; 0.29 I² = 0%

There was no evidence of effect of adherence interventions on systolic blood pressure (SBP), LDL, respiratory symptoms, hospitalization or osteoporosis symptoms [44] (Supplementary Figures S7–S28). SBP was judged to have a moderate certainty of evidence, while HbA1c, LDL, respiratory symptoms, hospitalization, and osteoporosis symptoms were judged to be of low or very low certainty (Supplementary Table S5).

### Secondary outcomes

Most studies did not report data on our secondary outcomes of patient and healthcare provider acceptability and satisfaction, and health equity impact. Five studies reported positive patient feedback regarding the intervention [43, 45-48]; four reported the intervention had no significant effect on patient satisfaction with their treatment or consultation [21, 22, 49, 50]; and one reported significantly higher patient satisfaction scores [44]. Three studies reported lower than expected take-up of the intervention in its entirety or an element of it [51-53], and in a study reporting qualitative reasons for this, these included preference to see their ongoing care provider, transport issues, and affordability [53].

Seven studies reported that healthcare providers found undertaking the intervention or associated training helpful, easy to use, time-saving, of appropriate workload, or useful in future [19, 21-23, 27, 45, 54]. One study reported that rates of provision of the intervention varied across locations, with lack of staff a barrier [53].

Few studies explicitly reported health equity impacts; the two that did so found similar impact of the intervention across different ethnicities [51, 55].

## Discussion

### Summary

Evidence of effect of consultation-based interventions on medication adherence was found only in certain disease subgroups, was of low or very low certainty, and was not robust to sensitivity analyses. There was no robust evidence of any impact of consultation-based interventions for medication adherence on clinical outcomes. There was some evidence that consultation-based interventions may improve medication adherence for patients with T2DM and chronic respiratory disease. However, the wide PIs imply that not all versions of the interventions may be beneficial in all populations and settings. Furthermore, the statistically significant results in these subgroups were not present in all sensitivity analyses, including those restricted to studies in which participants had low adherence at baseline, there was no robust evidence that adherence changes improved clinical outcomes, and evidence was lacking for consultation-based interventions improving adherence in other disease populations of primary or secondary cardiovascular disease prevention, or osteoporosis.

### Strengths and limitations

Strengths include the relevance and timeliness of this review focussing on interventions to improve health outcomes of chronic disease in time- and resource-constrained healthcare systems. Our search strategy was thorough, including databases, references, and citation searching, with no language constraints. The resulting sample is large with randomized trial data from over 26 000 participants from four continents, with varying healthcare systems but all interventions pragmatic and consultation-based, making the results more generalizable. We included only trials of interventions that were feasible in current primary care settings, and eligibility for the review was assessed by two independent assessors. We resolved assessor disagreements through consensus and followed PRISMA guidance throughout this study. The clinical outcomes of disease reviewed (SBP, HbA1c, LDL-C, measures of asthma, or COPD control) have been shown to correlate closely with end-outcomes including risk of major adverse cardiac events, hospitalization, exacerbation of chronic respiratory disease, and mortality [56-62]. The meta-analysis allowed a quantitative synthesis.

There were several limitations. First, we grouped studies by outcome measure only because the interventions had similar theoretical aims described by the PAPA. However, interventions varied greatly in their intensity and how they trained clinicians and intervention content, which may have led to the high observed heterogeneity. We made choices that aimed to maximize data availability. This meant that we included all studies, regardless of risk of bias and by choosing to combine studies with dichotomous and continuous outcomes using Chinn's method [40]. We addressed this through sensitivity analysis, which did not greatly change our estimates of effect.

Limitations of the evidence included in the review included that many studies included were assessed as being a high risk of bias; this was one of the potential contributing factors to heterogeneity we explored through sensitivity analysis. Many of the high risk of bias ratings were due to loss to follow-up, as missing data was unlikely to be independent from the outcome of medication adherence.

### Comparison with existing literature

This systematic review and meta-analysis is the first, to our knowledge, that focuses on medication adherence interventions for long-term preventative medications which are feasible to implement in routine healthcare consultations. Other reviews that included higher intensity interventions found that the diversity of clinical contexts and range of interventions trialled is a challenge to evidence synthesis and conclusions about what interventions to recommend in practice [36, 63]. A Cochrane review highlighted that many included trials had very complex interventions that would be difficult to implement in usual practice setting, with even effective interventions not leading to large improvements [36]. While this review explicitly did not focus on adherence interventions that may not be relevant or feasible for primary care clinicians, such as reducing prescription costs [64], fixed-dose drug combinations or “polypills” [65], reminder systems or messaging [66], or remote monitoring [67]; these may still represent useful strategies to improve medication adherence, depending on the healthcare system context. Alternative theoretical frameworks to PAPA regarding medication adherence, such as behavioural economics, may also support development of adherence interventions.

### Future implications

A decade on from the recommendations of the Cochrane review of medication adherence interventions, it remains the case that many studies of medication adherence use complex interventions which are not feasible in real-world practice. Future studies of behavioural interventions should include replicable description of the interventions and the behavioural change techniques used. Trial design should consider factors influencing scalability of interventions in primary care, such as technical innovation or training required and customizability of the intervention [12]. To ensure the validity and reliability of results of future studies on the topic of medication adherence, study teams should anticipate and try to minimize missing outcome data, such as by using independent measures of adherence including routinely collected prescription data.

## Conclusion

There was some low-certainty evidence that interventions delivered in consultation as part of routine care may improve medication adherence for patients with T2DM and chronic respiratory disease. However, there was no robust evidence that adherence changes improved clinical outcomes, and low-certainty evidence that such interventions do not improve adherence in other disease populations of primary or secondary cardiovascular disease prevention, nor osteoporosis.

The large number of studies excluded from this review based on our *a priori* feasibility considerations for implementation of interventions also demonstrated the capacity demands or system changes many interventions in the literature would require for successful implementation. Clearer reporting of staff capacity, capabilities, and time required to deliver interventions in both trials themselves and further systematic reviews on the topic of medication adherence would benefit both clinicians and policymakers.

## Supplementary Material

cmag007_Supplementary_Data

## Data Availability

The data underlying this article will be shared on reasonable request to the corresponding author.
