# Supplementary material for: Consultation-based interventions to optimize medication adherence in primary care: a systematic review
Source: Fam Pract. 2026 Mar 3;43(2):cmag007. doi: 10.1093/fampra/cmag007 (PMC13017097; doi:10.1093/fampra/cmag007)
Supplement: cmag007_Supplementary_Data [file cmag007_supplementary_data.pdf]

Supplemental File 1

Table of Contents

Glossary of terms 3
Search strategy 4
Table 1: Feasibility criteria 6
Outcome measures 8
Table 2: Adherence outcome measures in order of preference for extraction 8
Table 3: clinical outcomes in order of preference for extraction within clinical groups 8
Data synthesis methods 9
Supplemental Table 4: Characteristics of included studies 10
Risk of Bias 28
Traffic light plot of included Parallel Randomised Trials: 28
Traffic light plot of included Cluster-Randomised Trials: 29
Supplemental Table 5: GRADE assessment of certainty of evidence 30
Supplemental Figures 33
Supplemental Figure 1: Standardised mean difference in adherence, sensitivity analysis using HKSJ model 33
Supplemental Figure 2: Standardised mean difference in adherence, excluding studies at high risk of bias 34
Supplemental Figure 3: Standardised mean difference in adherence, limited to studies in which adherence was poor at baseline 35
Supplemental Figure 4: standardised mean difference in medication adherence, subgroup analysis by type of intervention 36
.... 36
Supplemental Figure 5: standardised mean difference in medication adherence, subgroup analysis by number of patient contacts required by intervention 37
Supplemental Figure 6: funnel plot for primary adherence outcome, studies colour-coded by disease group 38
Supplemental Figure 7: Mean difference in SBP, primary analysis using IVHet model 38
Supplemental Figure 8: Mean difference in SBP, sensitivity analysis using HKSJ model 39
Supplemental Figure 9: Mean difference in SBP, excluding studies at high risk of bias 40
.... 40
Supplemental Figure 10: Mean difference in SBP, limited to studies in which SBP was poorly controlled (as defined by study authors) at baseline 40
Supplemental Figure 11: funnel plot for SBP outcome 41
.... 41

Supplemental Figure 12: Mean difference in HbA1c, primary analysis using IVHet model 41
Supplemental Figure 13: Mean difference in HbA1c, sensitivity analysis using HKSJ model 42
Supplemental Figure 14: Mean difference in HbA1c, excluding studies at high risk of bias 42
Supplemental Figure 15: Mean difference in HbA1c, limited to studies in which HbA1c was poorly controlled (as defined by study authors) at baseline 43
Supplemental Figure 16: funnel plot for HbA1c outcome 43
Supplemental Figure 17: post-hoc sensitivity analysis for HbA1 outcome 44
Supplemental Figure 18: Mean difference in LDL, primary analysis using IVHet model 44
.... 44
Supplemental Figure 19: Mean difference in LDL, sensitivity analysis using HKSJ meta-analysis method 45
Supplemental Figure 20: Mean difference in LDL, excluding studies at high risk of bias 45
Supplemental Figure 21: Mean difference in LDL, limited to studies in which LDL was poorly controlled (as defined by study authors) at baseline 46
.... 46
Supplemental Figure 22: funnel plot for LDL outcome 46
Supplemental figure 23: Odds ratio of hospitalization, primary analysis using IVHet model 47
Supplemental figure 24: Odds ratio of hospitalization, sensitivity analysis using HKSJ model 47
Supplemental Figure 25: Standardised mean difference in respiratory symptoms, primary analysis using IVHet model 47
.... 47
Supplemental Figure 26: Standardised mean difference in respiratory symptoms, sensitivity analysis using HKSJ meta-analysis method 48
Supplemental Figure 27: Standardised mean difference in respiratory symptoms, limited to studies in which respiratory symptoms were poorly controlled (as defined by study authors) at baseline 48
Supplemental Figure 28: funnel plot for respiratory symptom outcome 49
PRISMA 2020 Checklist 50
References 53

Glossary of terms

CVD: Cardiovascular Disease
ED: Emergency Department
EHR: Electronic Health Record
FEV1: Forced Expiratory Volume in 1 second
GP: General Practitioner
HbA1c: glycated haemoglobin
HKSJ: Hartung-Knapp-Sidik-Jonkman
ICC: Intra-Cluster Coefficient
ICS: Inhaled Corticosteroid
LDL-c: Low density lipoprotein
MARS-5: Medication Adherence Report Scale, 5-item
MARS-A: Medication Adherence Report Scale for Asthma
MMAS-4, -8: Morisky Medication-Taking Adherence Scale, 4-item or 8-item
MI: Myocardial Infarction
MRA: Medication Refill Adherence
PAD: Peripheral Arterial Disease
PCI: Percutaneous Coronary Intervention
PDC: Proportion of Days Covered (by filled prescriptions)
PIL: Patient Information Leaflet
SBP: Systolic Blood Pressure
SD: Standard Deviation
SDM: Shared Decision-Making
SMD: Standardized Mean Difference
T2DM: Type 2 Diabetes Mellitus

Search strategy

We searched 5 databases from 2015 to 27th January 2025, these included Medline (OvidSP) [1946-present], PsycINFO(OvidSP) [1806-present], CINAHL(EBSCOHost) [1982-present] and Cochrane Database of Systematic Reviews& Cochrane Central Register of Controlled Trials via Cochrane Library, Wiley. In addition, we searched Europe PMC (https://europepmc.org/), ClinicalTrials.gov (https://clinicaltrials.gov/) and WHO International Clinical Trials Registry Platform(https://trialsearch.who.int/Default.aspx). We searched using subject headings and title/abstract/keywords for our main concepts or medication adherence, specific chronic conditions and RCTs. We limited the search to 2015 onwards, no other limits were applied. The results were saved to Endnote for storage and transferred to Covidence for deduplication and screening.
We also screened abstracts of all 811 included studies in two large systematic reviews (1,2), and 127 studies from citation searching.

Example search strategy for Medline:

1	Patient Compliance/
2	Medication Adherence/
3	((medication? or treatment or therapy or drug? or regimen or medicine?) adj2 (complian* or adheren* or noncomplian* or nonadheren*)).ti,ab,kf.
4	((statin? or antihypertensive? or anti-hypertensive? or anticoagula* or anti-coagula* or antiplatelet? or anti-platelet? or hypoglyc?emic? or inhaled corticosteroid? or inhaled glucocorticoid? or inhaled steroid? or metformin or aspirin or bisphosphonate?) adj2 (complian* or adheren* or noncomplian* or nonadheren*)).ti,ab,kf.
5	or/1-4
6	randomized controlled trial.pt.
7	controlled clinical trial.pt.
8	randomized.ab.
9	clinical trials as topic.sh.
10	randomly.ab.
11	trial.ti.
12	or/6-11
13	exp animals/ not humans.sh.
14	12 not 13
15	hypertension/ or essential hypertension/
16	myocardial ischemia/ or acute coronary syndrome/ or angina pectoris/ or coronary disease/ or coronary artery disease/ or coronary occlusion/ or coronary stenosis/ or myocardial infarction/
17	exp stroke/
18	atrial fibrillation/
19	Asthma/
20	Pulmonary Disease, Chronic Obstructive/

21	hyperlipidemias/ or hypercholesterolemia/ or hypertriglyceridemia/
22	Diabetes Mellitus, Type 2/
23	Heart Disease Risk Factors/
24	osteoporosis/ or osteoporosis, postmenopausal/
25	Osteoporotic Fractures/
26	((fracture? adj2 risk) or (fracture? adj prevention)).ti,ab,kf.
27	ischaemic heart disease.ti,ab,kf.
28	(cerebrovascular disease or cardiovascular disease or (cardiovascular adj2 prevention) or (cardiovascular adj2 risk)).ti,ab,kf.
29	((cardiovascular adj2 (medic* or drug)) or (cardiac adj2 (medic* or drug))).ti,ab,kf.
30	hypolipidemic agents/ or anticholesteremic agents/ or hydroxymethylglutaryl-coa reductase inhibitors/ or ((cholesterol adj2 (medic* or drug)) or statin?).ti,ab,kf.
31	hypoglycemic agents/ or biguanides/ or metformin/ or glycoside hydrolase inhibitors/ or dipeptidyl-peptidase iv inhibitors/ or sodium-glucose transporter 2 inhibitors/ or Glucagon-Like Peptide 1/ or Sulfonylurea Compounds/ or ((glucose lowering medic* or antidiabet* medic* or diabet*) adj2 medic*).ti,ab,kf.
32	anticoagulants/ or antithrombins/ or factor xa inhibitors/ or platelet aggregation inhibitors/ or (blood thinn* or warfarin).ti,ab,kf.
33	Antihypertensive Agents/ or angiotensin-converting enzyme inhibitors/ or angiotensin ii type 1 receptor blockers/ or Calcium Channel Blockers/ or (blood pressure adj2 medic*).ti,ab,kf.
34	(inhaled adj2 (corticosteroid? or glucocorticoid? or steroid?)).ti,ab,kf. or anti-asthmatic agents/
35	diphosphonates/ or bone density conservation agents/ or bisphosphonate?.ti,ab,kf.
36	or/15-35
37	5 and 14 and 36
38	limit 37 to yr="2015 -Current"

Table 1: Feasibility criteria

	Included	Excluded
**Change in patient care deliverable in consultation**	Change in care delivered as part of (face to face or remotely delivered; individual or group) patient consultation, including clinician education which has the intended effect of changing how they consult. (eg decision aid for use in consultation; patient counselling/motivational interviewing with or without provision of written information; or)<br><br>This could include provision of written information in addition to this consultation, but one-off provision of information (written/video/webpage) as a standalone intervention with no consultation component was excluded.	**Core part of** the intervention not deliverable as part of consultation eg<br>1. Prescription co-payment, voucher, free provision, or lottery schemes<br>2. Polypill, inhaler device, or dose schedule strategies<br>3. Interventions requiring additional equipment or remote monitoring (eg home blood pressure monitoring)<br>4. Peer- or family-member mentoring<br>5. Mobile app/text messaging/interactive voice recordings as core part of intervention<br>6. Intervention requiring additional investigations not usually requested from within primary care (such as coronary computed tomography, or genetic testing)<br>7. Comparison of follow-up delivered by primary versus secondary care<br>8. Stand-alone dose dispensing strategy eg pillbox, dose dispenser, dosette box without any in-consultation component.
**Interventionist**	Intervention which could delivered by staff in roles present in primary care multidisciplinary team (MDT) based on RCGP definition (3)	Intervention which would need to be delivered by staff in roles not present in usual primary care MDT (eg clinical psychologist, dietitian)

**Interventionist time required** (based on average time taken for actual delivery of intervention where described, otherwise based on planned time for intervention delivery; If no details given of average/planned contact time, decision based on inclusive estimates of consultation time of 15 minutes for each in-person contact, 10 minutes for each phone call, and 1 hour for each practitioner meeting/education session)	**If** time measured per patient, **both**:<br>1. 0-2 additional patient contact in addition to usual care<br>o No change in how calculated if group consultation<br>2. AND total additional time per patient of <=60 minutes over entire length of intervention<br>o For group consultations, the time per patient for the interventionist should be calculated as the session time/number of patients in the group -eg 6 minutes for a 1 hour session with 10 patients<br><br>If time measured per interventionist **only** (eg intervention is provision of provider education with intention this will change how routine consultations are delivered):<br>1. additional time in total per interventionist (not per patient) of <=1 working day/8 hours<br><br>If **both** patient-facing and non-patient-facing time required of interventionists, only the **patient-facing time criteria was assessed** for inclusion	More than these contacts

Outcome measures

If included studies reported more than one measure of behavioural medication adherence, the measure highest in the following order of preference was extracted, with continuous outcome data extracted in preference to dichotomous data for meta-analysis. If studies report multiple adherence outcomes in different drug classes, the primary adherence outcome as specified by study was extracted (or if the primary adherence outcome not specified, whichever is reported first).

Table 2: Adherence outcome measures in order of preference for extraction

Objective:	1. Medication electronic monitoring system (MEMS) data
	2. Electronic prescribing data such as PDC, MRA
	3. Pill count
	4. Blood or urine drug concentration
	5. Time in therapeutic range
Subjective:	6. Morisky Medication Adherence Scale
	7. Medication Adherence Recall scale
	8. Adherence to Refills and Medications Scale
	9. Brief Medication Questionnaire
	10. Score on other validated scale such as Hill-Bone compliance scale, medication subscale of Diabetes Self Management Questionnaire
	11. Number of doses reported as missed in a given time period

Table 3: clinical outcomes in order of preference for extraction within clinical groups

Prevention of CVD	1. Systolic Blood Pressure (single or average of multiple readings)<br>2. LDL-C<br>3. Major Adverse Cardiac Events (MACE)<br>4. Disease-specific hospitalisation or death
Established CVD	5. Systolic Blood Pressure (single or average of multiple readings)<br>6. LDL-C<br>7. Major Adverse Cardiac Events (MACE)<br>8. Disease-specific hospitalisation or death
Type 2 Diabetes	1. Hba1C<br>2. Disease-specific hospitalisation or death
Respiratory disease	1. Validate respiratory symptomatology questionnaire eg Asthma control test score<br>2. Exacerbations of asthma<br>3. Exacerbations of COPD<br>4. Disease-specific hospitalisation or death
Osteoporosis	1. Bone density measurement (DEXA Z-score, bone turnover blood test marker)<br>2. Fracture

Data synthesis methods

For outcomes in which the same effect measures were reported across studies (blood pressure, LDL cholesterol, and HbA1C), change scores (adjusted for clustering where appropriate, and adjusted for other factors if reported) were meta-analysed and presented as mean difference. Where change scores were not reported by the authors, these were imputed from the reported baseline and endpoint scores. A correlation coefficient of rho=0.5 was used, which was more conservative than the correlation coefficient calculated from the one included paper ((4); rho=0.28) in which direct calculation of a correlation coefficient was possible.

For outcomes in which different effect measures were reported across studies (medication adherence, respiratory disease symptom scores), standardized mean difference was calculated at endpoint. For dichotomous effect measures of these outcomes, odds ratio was calculated from reported endpoint data, and converted to SMD by the Chinn formula (5). Where available, the standard error of the odds ratio was calculated using reported p value, or otherwise SE of the odds ratio was calculated using the cci function in Stata version 14.

For continuous effect measures, SMD was calculated as the mean difference at endpoint, divided by the SD of the control group at endpoint wherever available (the SD of the control group at endpoint was used due to adherence not always being measured at baseline, for example in trials where medication was newly started). Where the SD of the control group at endpoint was not reported, SD of the control group at baseline was used to calculate the SMD, and where this was not available, pooled SD of the whole study population at baseline was used.

In studies with multiple intervention groups of the same type (but not necessarily same intensity) we pooled intervention groups by fixed effect meta-analysis. In studies with multiple intervention groups of different types, we treated intervention groups separately by comparing each intervention group against subsets of the control group to ensure double comparison was avoided.

Where multiple results or time points were presented compatible with each outcome domain, results were extracted at 12 months or the longest available follow-up if shorter than 12 months; and outcome measures used in meta-analysis based on *a priori* criteria, (tables 2 and 3, supplemental file 1).

For cluster trials, results adjusted for clustering were extracted, where available; otherwise, we extracted results and adjusted for clustering by calculating a design effect using an ICC reported in the same or other similar trials.

Results were converted where necessary (for example, where higher adherence is indicated by either higher or lower scores in different studies) to ensure consistent directionality of study outcomes within meta-analysis. For statistically significant results, we back-converted SMDs to values on the original scale by multiplying by suitable illustrative standard deviation values (6).

Supplemental Table 4: Characteristics of included studies

Study	Prevention area	N random-ised	Main inclusion criteria	Main exclusion criteria	Relevant outcomes assessed	Length of follow-up	Groups	Content
**Perceptual**								
Buhse 2018<br><br>cRT<br><br>Germany	T2DM	279 in 22 clusters	-between 40 and 69 years<br>-diagnosed with type 2 diabetes<br>-HbA1c levels of <9%	History of:<br>-ischaemic heart disease<br>-stroke<br>-proliferative retinopathy<br>-CKD3	-Medication adherence (assessed by blinded interviewer using standardised interview guide in comparison with documented prescriptions)<br><br>-HbA1c	6 months	Intervention<br><br>Change in care delivered by medical assistants and GPs	1). Provider training on shared decision making for GPs and medical assistants, didactic lectures and role plays lasting 4-5 hours<br>2) patient teaching module (medical assistant): 4-6 patients, 90 mins session. At the end of the teaching session, patients documented their preferences on treatment goals and asked to document their personally most important treatment goal<br>3) consultation with GP 1 week after teaching session, 10 minutes; discussion of patient decision aid and individual treatment goals; Informed Shared Decision-Making Program consultation guideline to structure the conversation
							Control	Minimal counselling
Cooper 2011<br><br>cRT<br><br>USA	Primary CVD prevention	279 patients, 50 physicians	Physicians: seeing patients at least 20 hours per week at a participating site<br><br>Patients:<br>- aged >=18 years<br>-diagnosis of hypertension (at least one claim with the ICD-9 code 401 in the preceding year).	Physicians: intending to leave practice within 12 months.<br><br>Patients: too ill/disoriented/unresponsive to complete baseline assessment, those with eg AIDS/HIV, schizophrenia, cance, dementia; end-stage renal disease, congestive heart failure, or active tuberculosis	-Medication adherence (Morisky 4-item scale)<br><br>-SBP	12 months	Physician Intensive/ Patient Minimal Intervention:<br><br>Change in care delivered by family physicians or general internists	Physician communication skills program (described in protocol as taking 2 hours to administer), including interview with simulated patient and review of this; completion of workbook exercise; receiving copy of HTN guidelines.
							Physician + Patient Minimal Control	Minimal training and minimal counselling
							Other intervention arms in study did not meet inclusion criteria	
Ebid 2022	T2DM	125	Aged 18+<br>- T2DM	-eGFR <30ml/min<br>-hepatic disease	-Medication adherence	6 months	Intervention	Patients met with clinical pharmacist for 30 mins after seeing physician; pharmacist

RT<br><br>Egypt			- managed with diet or oral hypoglycaemic agents	- on insulin,<br>-pregnant or breastfeeding<br>- dementia/cognitive impairment<br>- severe/unstable CCF that has required hospitalization<br>- endocrine disorders affecting glucose metabolism eg thyroid dysfunction<br>- those 'unable to provide a consent form'	(Morisky Green 25-item)<br><br>-HbA1c		Change in care delivered by pharmacist	provided patient education and counselling including types, doses, and side effects of medication, complications, importance of medication adherence and self-management of glucose testing at each visit. Also gave physicians recommendations on drug therapies including dose titration/adding/changing agents.
							Control	Usual care
George 2019<br><br>cRT<br><br>USA	Respiratory disease	80	-Adults >= 18<br>-self-identifying as Black/multiracial<br>- prescribed ICS in preceding 12 months<br>-uncontrolled asthma, and erroneous beliefs about asthma management/treatment.	-Participation in focus groups used to inform intervention development<br>-comorbid disorders that would preclude longitudinal data collection.	-Medication adherence (MARS-A)<br><br>-Asthma control (ACQ)	3 months	Intervention	Semi-scripted SDM session using motivational interviewing techniques. Clinicians trained to do 4 steps: 1) raising the subject of ICS non-adherence and asthma control; 2) making an explicit connection between non-adherence and uncontrolled asthma; 3) enhancing motivation to increase ICS use by exploring and resolving ambivalence towards treatment; and 4) engaging in SDM. Delivered in mean 9:11 minutes; range 4:10–15:50.
							Attention control	Unscripted discussion of healthy lifestyles, 70% consisted of discussion of nutrition and exercise
Goeman 2013<br><br>RT<br><br>Australia	Respiratory disease	124	- age 55 years or older<br>-asthma (as confirmed by baseline lung function tests)	- COPD (as determined by lung function tests +smoking history)<br>- inability to participate in interactive education session due to language or cognitive issues	-Medication adherence (smart inhaler)<br><br>-Asthma control (ACQ)	12 months	Intervention	Person-centred education tailored to address issues raised by participants' responses to a Patient Asthma Concerns Tool designed for this study, and self-management education
							Control	Minimal counselling
Goni 2024<br><br>RT<br><br>Australia	Established CVD	29	-admitted primarily due to an MI diagnosis<br>-at least 18 years old<br>Exclusion criteria were defined as	-those who developed an NSTEMI or STEMI during an existing admission for an alternative reason<br>-had a documented psychiatric illness (excluding depression due to the frequent prevalence post-MI)<br>- hearing	-Medication adherence (MMAS-8)<br><br>-Hospital readmission related to cardiovascular health, including Medication, over 12 weeks		Intervention	One individualised education session 4-6 weeks after discharge, 40 mins.<br>Intervention interview questions aimed to address non-adherence and comprehension of the medication plan. After assessing the underlying causes, the pharmacist provided individualised support to increase adherence and/or comprehension of the treatment plan. Teach-back technique was utilised to confirm understanding.
							Control	Not described

				impairment as the intervention occurred via telephone				
Jaspers 2021<br><br>RT<br><br>Netherlands	Established CVD	303	- referred to study centre for CVD screening,<br>-Age between 45-80<br>-with CVD (coronary arterial disease, cerebrovascular disease, peripheral arterial disease, or abdominal aortic aneurysm)<br>- current statin use	- terminal malignancy<br>-not returning baseline questionnaires	-Medication adherence (BMQ)<br><br>-LDL-c	6 months	Individualised CVD-free Life Expectancy	Standard practice plus provision, and structured telephone discussion of, leaflet with 'personalised health profile' explaining effects of continuing with current statin/discontinuing statins/intensifying statin therapy- communicated in terms of CVD-free life expectancy. Also strongly encouraged to visit their GP within 2 weeks to discuss the received information
							Individualised 10-year absolute risk<br><br>(Intervention arms pooled for meta-analysis)	Standard practice plus provision, and structured telephone discussion of, leaflet with 'personalised health profile' explaining effects of continuing with current statin/discontinuing statins/intensifying statin therapy- communicated in terms of 10 year CVD risk. Also strongly encouraged to visit their GP within 2 weeks to discuss the received information
							Control	Standard practice
Karagiannis 2016<br><br>cRT<br><br>Greece	T2DM	215 patients, 9 clusters	-adults who have had T2DM for more than a year<br>-speak Greek,<br>-Hba1c between 7.5 and 10%<br>-required treatment intensification and had more than one options available as judged by their clinicians,<br>-agreed to be followed for at least 24 weeks by the participating clinician	None stated	-Medication adherence (PDC)<br><br>-HbA1c	5.5 months	Intervention	Use of decision aid by physician during consultation, consisting of seven cards that simply display the benefits and harms of commonly used antidiabetic medication classes across domains that patients and clinicians often consider important when choosing among several treatment options (reduction in HbA1c, weight change, hypoglycaemia, main adverse effects, treatment cost, daily sugar testing and drug-related daily routine). During the consultation, the patient is presented with the seven cards by the physician and is asked which of the cards they would prefer to discuss. Ideally, after reviewing the chosen cards and discussing about alternative medication options, both the patient and the physician arrive at the medication choice that best matches the patient's preferences.
							Control	Usual care
Kung 2009<br><br>RT	Osteoporosis	596	- Ambulatory postmenopausal women <=85<br>-naïve or lapsed or current bisphosphonate users	-Hypersensitivity to bisphosphonates<br>-not able to stand/sit upright for at least 60 min<br>-not able to swallow tablet	-Medication adherence (self-report)	6 months medication adherence	Intervention	Received feedback at 3 months on serum carboxy-terminal collagen crosslinks (CTX) result, which is a marker of bone turnover and show large changes in response to antiresorptive treatment within a few months

Hong Kong, Indonesia, Philippines, Taiwan and Thailand				-taking drugs that may interfere with BMD within 30 days prior to 1st dose study drug (selective estrogen receptive modulators/anti estrogen/aromatase inhibitors/calcitonin/oral corticosteroids/Hormone Replacement therapy)	-Patients presenting with osteoporosis symptoms	12 months osteoporosis symptoms	Control	Usual care; did not have CTX result fed back to them at 3 months
Kunneman 2022<br><br>cRT<br><br>USA	T2DM	495	-Clinicians in participating practices caring for patients with T2DM<br>-Adults with T2DM<br>- Hba1c in the last 12 months of >7.3%<br>- not receiving insulin<br>-with a prescheduled appt with a primary care clinician participating the study<br>-available for 12m follow-up	- 'intellectual or sensorial barriers to providing written informed consent'	-Medication adherence (PDC)<br><br>-HbA1c	12 months	Intervention	Use of the Mayo Clinic Diabetes Medication choice conversation aid (https://diabetesdecisionaid.mayoclinic.org/)during clinical encounter, which presents general considerations and adverse effects of diabetes medication, organized in terms by topics that matter to patients: weight change, daily routine, blood sugar levels (HbA1c), daily blood sugar testing, hypoglycaemia, and cost; patient and clinician negotiate which cards to review, in what order, and in what detail, until they arrive at a preferred approach by consensus.
							Control	Usual care
Manze 2015<br><br>cRT<br><br>USA	Prevention of CVD	379	-patients aged >= 21<br>-diagnosis of hypertension<br>-prescribed at least one antihypertensive medication<br>-self-identified as white or black race		-Medication adherence (Hill-Bone Compliance to High Blood Pressure Therapy scale)<br><br>-SBP	9 months adherence<br><br>5 months clinical	Intervention	-Primary care providers given two workshops: one on patient-centred counselling, to identify barriers to adherence and strategies for addressing them using 5As framework; role plays.<br>- Second workshop intended to improve providers' cultural competency, for providers to understand patient's conceptualization of their illness, exploring patients' concerns and addressing these, and getting patients to playback understanding of instructions, and providing patients written instructions.<br>- Pop up reminders in EMR to remind intervention providers to counsel patients using this approach, copies of counselling algorithm placed in consultation rooms included suggested scripts.
							Control	Usual care
Mullan 2009<br><br>cRT	T2DM	85	Clinicians: doctors, PAs, NPs treating adults with T2DM in primary care	-Well or very poorly controlled DM (HbA1c <7% or >9.5%)	-Medication adherence (7-day total recall)	6 months	Intervention	Consultation using decision aid of cards showing advantages/disadvantages of adding different options of hypoglycaemic medication:

USA			Patients:<br>- Adults with T2DM diagnosed for at least 1 year<br>-HbA1c tests conducted less than 6 months prior to enrolment and results between 7.0% and 9.5% while taking 3 or fewer antihyperglycemic medications<br>-not using insulin	-taking insulin	-HbA1C			clinician intended to show 6 cards that describe the possible effects of the medications on 6 outcomes: “Weight Change,” “Hypoglycaemia” ,“Blood Sugar”, “Daily Routine,” “Daily Sugar Testing,” and “Side Effects” to patient before asking them which area to discuss first, and discussing all needed to come to decision suiting patient's circumstances. Patient given take home pamphlet showing same information.
							Control	Usual care
Powers 2011<br><br>RT<br><br>USA	Prevention of CVD	89	- age >=55<br>-diagnosis of hypertension<br>-received a prescription for antihypertensive in the past year<br>- had SBP >140 or DBP >90 on most recent blood pressure measurement within the last 12 months<br>-and had an electrocardiogram within the last 5 years to evaluate the absence or presence of left ventricular hypertrophy<br>-enrolled in primary care clinic for at least one year	-hospitalized for a myocardial infarction or coronary artery revascularization or had a diagnosis of metastatic cancer in the past 6 months<br>-history of stroke<br>-active diagnosis of psychosis or dementia documented in medical record<br>-participating in another chronic disease self-management study<br>-resident of a nursing home<br>-no access to a telephone	-Medication adherence (MMAS-4)<br><br>-SBP	3 months	Intervention	-Consultation in which information based on their Framingham CHD and stroke risk score was presented to patients verbally and in graphic form with bar charts representing patient's risk. Presented with potential strategies to improve risk through medication and lifestyle factors. Also received written PIL on risk of MI/stroke (same as standard arm).
							Control	Minimal counselling (written PIL on risk of MI/stroke)
Sarycheva 2017<br><br>RT<br><br>Russia	Primary CVD prevention	150	-Age 40-65<br>-hypertension (SBP >140 and/or DBP >90)<br>and<br>-total cholesterol >5mmol/l and/or LDL >3mmol/l and/or HDL <1mmoll for mean and <1.2 mmol/l for women and/or triglycerides >1.7mmol/l.<br>and	- Established coronary artery disease<br>-clinical atherosclerosis<br>-diabetes mellitus<br>-heart defects<br>-other chronic disease including cancer	-Medication adherence (MMAS-4)<br><br>-SBP<br><br>-LDL	12 months	Intervention	Visual demonstration of change in personal risk if targets were achieved in blood pressure, cholesterol, smoking cessation, weight loss.

			-With inadequate control of hypertension before the study<br>and<br>-failure to control dyslipidaemia by diet. And<br>-target organ damage: (microalbuminuria, left ventricular hypertrophy, subclinical atherosclerosis of the carotid arteries)				Control	Standard care (including recommendations on a healthy lifestyle) but no visual demonstration of risk.
Tinsel 2013<br><br>cRT<br><br>Germany	Multiple	1120	For GPs:<br>-in Southwest Germany<br>-offering full range of family health care services<br>-not participating in another study implementing any SDM training.<br><br>Patients:<br>-repeated prescription of antihypertensives<br>-aged 18<br>- with medical insurance<br>-understanding German<br>-at least one relevant comorbid disorder (DM, CHD/MI, stroke/TIA, peripheral arterial disease)	For patients:<br>-dementia<br>-mental handicap<br>-short life expectancy	-Medication adherence (MARS)<br><br>-SBP	12 months	Intervention	GPs took part in 6-hour shared decision-making training programme to guide their usual consultation, covering<br>1) information on hypertension<br>2) physician-patient communication and risk communication<br>3) the process steps of SDM<br>4) motivational interviewing;<br>5) introduction of a decision table listing options to lower cardiovascular risk<br>6) use of case vignettes for role plays simulating physician-patient consultation<br>7) recommendation of use of cardiovascular risk calculator in consultation, which included elements of SDM
							Control	Usual care. No training, reminders, or materials given to providers
**Combined**								
Alfian 2021<br><br>cRT<br><br>Indonesia	Multiple	113 patients; 10 clusters	->=18 years old<br>-diagnosed with T2DM for at least 1 year based on the patient's medical record<br>-using at least one antihypertensive drug in the last 3 months<br>-suboptimal medication adherence to antihypertensive drugs (MARS-5 score <20/25).	- severe mental or physical constraints<br>-pregnancy or in the lactation period<br>-illiterate in the Indonesian language<br>-enrolled in another intervention study<br>-those not responsible for taking their own medication were excluded.	-Medication adherence (MARS)<br><br>-SBP	3 months	Intervention	-The pharmacist discussed patient-specific barrier(s) for medication adherence (consultation lasting average 14 mins). Simple question-based flowcharts and the adherence intervention wheel were provided to support the pharmacy staff in identifying the patient's personal adherence barriers and tailoring the intervention to these barriers: we defined four main adherence barriers that could be addressed by the community pharmacists: (1) forgetfulness; (2) lack of knowledge; (3) lack of motivation; and/or (4) other drug-related problems. The intervention strategy was tailored to the identified adherence barrier(s),

								and patients were involved in goal setting and writing the agreed goal(s) at the top of a personalized leaflet.<br>-A follow-up session was conducted 1 month after the baseline session, as part of medication refill (average 11 minutes). The short-term effect of the intervention was evaluated (MARS), and nonadherence problems that had not been addressed during the first session were discussed. Based on patients' responses to the MARS, those who had already become adherent by the follow-up session were complimented and asked about their expectations to maintain good adherence. In patients that still showed nonadherence, the pharmacist, together with patients, made changes to the action plan and discussed additional interventions. This session was again ended with involving patients in goal setting and writing the agreed goal(s) at the top of a personalized leaflet.
							Control	Minimal counselling
Armour 2007<br><br>cRT<br><br>Australia	Respirato ry disease	396; 57 clusters	Pharmacies:<br>-Quality Care Pharmacy Programme accreditation<br>-availability of a computer system compatible with the<br>spirometer software to be used in the study<br>-ability to attend training sessions<br>-a minimum of two pharmacists on duty at any one time.<br>Patients:<br>-aged 18-75 with asthma	For pharmacies:<br>- current involvement in any other research project. For patients: terminal illness, currently enrolled in another trial, don't self-admin their inhaler, don't speak English well enough to complete questionnaires independently	-Medication adherence (BMQ)<br><br>-Asthma severity (assessed using National Asthma Council of Australia severity scoring tool)	6 months	Intervention	Targeted counselling and education on asthma medication and triggers, review of inhaler technique, adherence assessment, detection of drug-related problems; goal setting and review; referral to GP if needed. (1-2 sessions maximum, mean 1.27)

			- One or more in the previous four weeks of: use of reliever medication >3 times per week; waking at night with asthma symptoms on at least on occasion; time off work due to asthma; asthma symptoms at least once per week<br>-no visit to doctor for asthma for last 6 months				Control	Usual care
Calvo 2021<br><br>RT<br><br>Spain	Established CV disease	143	-undergoing PCI for MI<br>-aged over 75	-Patients unable to perform geriatric assessment due to inability to answer the questions by themselves<br>-Patients admitted to nursing homes or social health centres	-Medication adherence (composite dichotomous outcome based on maximum score on MMAS-4, all medications collected, between 80-110% on Haynes Sackett score, and attendance at all scheduled visits<br><br>-Rehospitalization rate	12 months	Intervention	1) Consultation with registered nurse 3 months after PCI, covering review of medication and nurse health education to increase therapeutic adherence (which could include explanation; discussing with cardiology to minimise medications; making a calendar card to visualise dose schedule; and discussing pharmacist provision of dosette box)<br>2) Reminder call at 6 months; patient asked about their current drugs and if they had any questions about them, and the importance of therapeutic adherence stressed again.
							Control	Not described
Choudhry 2018<br><br>cRT<br><br>USA	Multiple	4078 patients, 14 clusters	- ≥18 and <85 years of age<br>- receiving care from an Atrius primary care provider and who were insured by one of 4 large health insurers with whom Atrius had risk-sharing contracts<br>-diagnosis of hyperlipidaemia, hypertension or diabetes based on having filled a prescription for a medication used to treat one of these conditions	-less than 6months of continuous enrolment in the health plan (to allow adequate assessment of eligibility)<br>-no available telephone contact information	-Medication adherence (PDC)<br><br>-SBP<br><br>-HbA1C<br><br>-LDL	Adherence:<br><br>BP: 3.2 months<br><br>HbA1C: 12 months<br><br>LDL: 7.5 months	Intervention	1) Individually tailored telephone consultation conducted by a staff clinical pharmacist using brief negotiated interviewing technique to identify barriers to adherence and negotiate a plan to improve adherence and disease control (which could include counselling about benefits and risks of treatment, recommendation of reminder aids, suggestion of medication timing change, referral to social work, and strategies to reduce adverse effects appropriate to the patient's circumstances)<br>2) Mailed progress reports were sent to intervention patients at 6 and 9months after randomization on behalf of their primary care clinician and summarized personalized and updated information about

			-evidence of poor or worsening disease control for at least one of these conditions (based on most recent lab/BP value in EHR, based on clinical guideline targets established by the Eighth Joint National Committee (JNC 8), the American Diabetes Association, and the American Heart Association/American College of Cardiology for hypertension, diabetes and cholesterol, respectively)<br>- less than 80% adherent to these medications measured with PDC					disease control generated using data from the electronic health record and medication adherence from administrative claims.
							Control	Usual care
Duncan 2020<br><br>cRT<br><br>USA	Established CVD	6024 patients, 40 clusters	-Hospital eligible if their ED treated stroke patients<br>-Adults diagnosed with ischaemic stroke, haemorrhagic stroke or TIA and discharged directly home<br>-English or Spanish speaking.	-Subdural or aneurysmal haemorrhage.<br>-Excluded from analysis if had subsequent stroke events within study period	-Medication adherence (MMAS-4)<br><br>-90-day mortality	3 months	Intervention	1) telephone follow-up within 2 business days of hospital discharge<br>2) a clinic visit targeted to occur 7 to 14 days post-discharge. Standardized clinical assessments that included social and functional determinates of health informed an individualized care plan discussed and patient materials delivered of at the point of care, incorporating education, secondary prevention, rehabilitation, recovery, referrals to community-based resources, and caregiver support services. Key messages included: Know your Numbers (eg, BP, HbA1c), Engage Mind and Body (eg, treat depression, engage in rehabilitation), Willingness (eg, self-manage modifiable risk factors, manage medications), and Support (eg, seek community support services).
							Control	Usual care hospitals continued their current standard of post-acute care. Patients in both arms received a study brochure and information on BP control and were advised to track their BP. Patients received materials to enhance retention, including mailings and a $10 incentive for survey participation.

Farmer 2012<br><br>RT<br><br>UK	T2DM	211	- aged >=18 years<br>-type 2<br>diabetes of at least three months duration<br>-able to give<br>informed consent<br>-currently taking any oral glucose lowering<br>agent<br>-with a HbA1c ≥7.5% (58mmol/mol).<br>-deemed by their general practitioner to<br>be appropriate for tight glycaemic control and independent<br>in medication taking.	- 'co-morbidity'	-Medication adherence (MEMS)<br><br>-HbA1c	4.6 months	Intervention	Clinic visit with nurse; 30 mins intervention.<br>1) motivational component of the intervention: the nurse elicited patients` beliefs relevant to their intention to take medication regularly as prescribed using a series of questions based on the Theory of Planned Behaviour. These perceived benefits and harms of taking medicines, views of other people who were important to them and factors that may facilitate or inhibit taking medicines regularly as prescribed. Positive beliefs were reinforced verbally and non-verbally through provision of tailored information and problem solving was facilitated around negative beliefs.<br>2) action planning component: the nurse asked patients to generate and write down the exact circumstances in which they would take their medication (using an "if-then" formulation to elicit where, when and how this would occur).
							Control	Usual care
Foster 2014<br><br>cRT<br><br>Australia	Respirato ry disease	143	GP inclusion criteria: access to computer and e-mail,<br>-not currently participating in another adherence-promoting study. To minimize cross-contamination between intervention groups, only 1 GP from a practice could participate.<br>Patients:<br>-Aged 14 to 65 years<br>-suboptimal asthma (ACT <=19)<br>-prescribed twice daily ICS/LABA for 1 month or more.	- Asthma exacerbation (oral steroids/ED/hospitalization) in the last month<br>-MART combination therapy<br>-major resp disease eg COPD<br>-serious uncontrolled medical conditions<br>-clinically important visual or auditory impairment<br>-shift workers with a variable roster<br>-pregnant or lactating women	-Medication adherence (smart inhaler electronic monitoring)<br><br>-Asthma control (ACT)	6 months	'Personalised Adherence Discussions' Intervention	1) GPs were trained (2 hours of training) to carry out a personalized discussion (duration not described) about the patient's key barrier(s) to adherence and to help the patient set goals and goal-achievement strategies around an asthma issue that the patient wished to resolve, using patient-centred materials, eg GPs asked patients to complete a short questionnaire about barriers to controller inhaler use at the beginning of discussion.<br>2). The GPs were prompted to use their practice software or post-it notes to facilitate following up at any subsequent visits on issues identified during these discussions. At the follow-up visit, the GP was asked to review the patient's goals and strategies. The GP and the patient could also choose to continue the previous discussion topic, or to discuss a different adherence barrier
							Control	'Active usual care' based on government-incentivised primary care asthma management programme including provision of written asthma action plan, inhaler technique review/education, and follow-up appointment.

							Other intervention arm did not meet review inclusion criteria	
Hesselink 2004<br><br>RT<br><br>The Netherlands	Respiratory disease	276	-Patients aged 16-75<br>-with diagnosis of asthma/COPD/mixed disease<br>-Current use of asthma or COPD medication<br>-experience of disease symptoms in the past year	- other specific pulmonary or terminal diseases	-Medication adherence (3-question checklist)<br><br>-Respiratory symptomatology (Medical Research Council dyspnoea scale)	12 months	Intervention	Semi structured 30 min consultations with GP assistant in the first year of the study, on a) information about disease, medication, compliance; b) control and inhalation technique; c) barriers in coping with the disease d) smoking cessation offer e) advice on when to consult doctor. Free booklets (on eg use of medication or dealing with allergy)
							Control	Usual care
Jarab 2012<br><br>RT<br><br>Jordan	Respiratory disease	133	- patients only attend the outpatient COPD clinic at the Royal Medical Services<br>-confirmed diagnosis of COPD by the hospital consultant for at least 1 year<br>-over 35 years old<br>-FEV1 30–80% of the predicted normal value --hospital consultant agreement that the patient is suitable for entering the trial	-moderate to severe learning difficulties<br>-mobility problems<br>-confusion, disorientation<br>-terminal illness<br>-congestive heart failure<br>-attended a pulmonary rehabilitation programme or had consulted a pulmonary nurse or clinical pharmacist in the last 6 months.	-Medication adherence (MMAS-4)<br><br>-Respiratory symptomatology (St George's Respiratory Questionnaire)	6 months	Intervention	A structured patient education about COPD and management of its symptoms was delivered by the clinical pharmacist for the intervention patients in a separate room at the outpatient clinic. The clinical pharmacist also completed a medication table designed specifically to discuss types, indications, doses, frequency of administration, and possible side effects for each prescribed medication. Furthermore, the importance of simple exercises, symptoms control and the technique for expectoration were discussed with the intervention patients and a booklet on these provided. The clinical pharmacist used motivational interviewing technique with the aim of improving adherence to the prescribed treatment. Patients who still smoked were referred to a special smoking cessation programme within the hospital
							Control	Not described
Lyons 2016<br><br>RT<br><br>UK	Multiple	677	- Patients prescribed at least one oral medication for type 2 diabetes and/or lipid regulation<br>-getting this dispensed by pharmacy2u.	- Living outside England<br>-age <18	-Medication adherence (Diagnostic Adherence to Medication Scale questionnaire)<br><br>-HbA1c	6 months	Intervention	Two telephone consultations with a pharmacist, 4–6 weeks apart. The telephone consultations followed a semi-structured condition-specific interview guide, aiming to identify any particular problems or concerns that the patient may be having, targeting issues related to medication adherence. The pharmacist recorded any problems or concerns identified, and interventions provided, on a specially designed page on the pharmacy database. If the patient identified no particular issues, the pharmacist reinforced the importance of continuing to adhere

								to the prescribed medication regimen and offered healthy living advice.<br>2) The follow-up phone call offered an opportunity to review any issues discussed in the initial consultation and to identify any new or outstanding problems to be addressed.<br>3) At the end of the telephone consultation each participant was posted a letter summarising the key points discussed, and a personalised list of their current prescribed medications, known as a medicines reminder chart, indicating what each medicine is for, and how much of, and when, each should be taken
							Control	Usual care; no specific pharmacist consultations but patients had the ability to contact the pharmacist if they wished to.
Manfrin 2017<br><br>cRT<br><br>Italy	Respiratory disease	1263 pharmacies, 360 clusters	Pharmacists:<br>- qualified and registered with the Italian Pharmacy Board practising in Italy<br>-working in pharmacies with private consultation facilities and internet connection.<br>-at least one year of experience in providing advice to patients;<br>-already provide one or more services such as blood pressure monitoring in order to demonstrate	Pharmacists:<br>-currently involved in any other clinical pharmacy research project.<br>Patients<br>- terminal illness as identified by the pharmacists through the prescription coding<br>-currently enrolled in another clinical trial<br>-do not self-administer their inhaler<br>-are not able to communicate well in Italian both written and spoken	-Medication adherence (2 questions adapted from MMAS-8)<br><br>-Respiratory symptomatology (ACT)	3 months	Intervention	A systematic, structured interview, conducted in a private room within the pharmacy, which covered asthma symptoms, medicines used, attitudes towards medicines and adherence. The pharmacists were trained to identify pharmaceutical care issues (PCIs) which could impact on optimal medicines use or asthma control and provide advice to the patients and recommendations to their GP, as necessary.

			advanced consultation skills and experience<br>-able to attend training sessions.<br>Recruited pharmacists asked to recruit five patients with<br>- at least 18 years of age<br>-diagnosed with asthma for at least six months before enrolment to the study<br>-have a prescription(s) for asthma medication or drugs for obstructive airways disease.				Control	Usual care (delayed receipt of intervention)
Nguyen 2018<br><br>RT<br><br>Vietnam	Established CVD	166	-Patients admitted for unstable angina or MI who survived inpatient stay	-Already participated in a medication adherence study<br>-discharged without prescription<br>-'considerable' cognitive impairment<br>-unable to communicate in Vietnamese<br>-unable to identify own medications<br>-could not provide a telephone number<br>-stayed in hospital three days or less	-Medication adherence (MMAS-8)<br><br>-Hospital readmission	3 months	Intervention	1) First pharmacist counselling session of 30 mins including information on ACS, risk factors, and prevention; assessment of past experience using medication, encouragement and tailored advice; provision of drug leaflets and pill organizer; teach back and correcting misunderstanding.<br>2) Second session of 30 min telephone counselling within 2 weeks after discharge including assessment of general and medication-related issues; encouragement and tailored advice; teach back and correcting misunderstanding
							Control	Usual care post-acute coronary syndrome (which involved appointments every 2- 4 weeks)
Noseworthy 2022<br><br>RT<br><br>USA	Prevention of CVD	922	-Adults<br>-nonvalvular AF<br>-CHADSVASC score of 1+ in men or 2+ in women	-mechanical heart valves<br>-prior left atrial appendance occlusion device implantation<br>-moderate/severe rheumatic mitral stenosis	-Medication adherence (PDC)<br><br>-Cerebrovascular events	10 months	Intervention	Consultation using<br>1) anticoagulation choice tool risk calculator to calculate personalised risk of stroke at 1 and 5 years<br>2) issues cards that support patient-clinician conversation on important factors that may affect choice of agent and patient ability to adhere (eg diet, recreational activities, travel).

							Control	Usual care
O'Carroll 2013<br><br>RT<br><br>UK	Establish ed CVD	62	-Patients experiencing first stroke or TIA<br>-discharged home<br>-on any preventive stroke medication	- <13 on Frenchay aphasia screening tests or <23 on MMSE<br>-not responsible for own medication<br>-not on any antihypertensive medication<br>-using pharmacy supplied dosette box	-Medication adherence (MEMS)<br><br>-SBP	3 months	Intervention	1) First consultation with doctor in which patients were asked to make a plan to link taking tablets to something else they did every day, and write down time, place, and linked activity on an individualised worksheet. Patients asked to repeat plan up to 3 times until they could remember it without reading it.<br>2) Second consultation: effectiveness of implementation plan and any barriers was reviewed, and any required changes developed collaboratively. Beliefs and illness and medication elicited based on BMQ and BIPQ and any mistaken beliefs challenged. Aimed to increased necessity beliefs and decrease concern beliefs, eg by informing them of current guidelines based on risk reduction, and informing them on likelihood of experiencing side effects.
							Control	Attention control
O'Connor 2014<br><br>cRT<br><br>USA	Multiple	2778	-age 18-75<br>-EHR indicators of diabetes<br>-prescribed a new class of medication (not filled in the past 180 days) for HbA1c/BP/LDL<br>-Hba1c >=8%, SBP >= 140 mmHg, or LDL >=100mg/dL at the time of prescription<br>- receiving care at a participating centre for at least 15 months before enrolment	- less than 6 months of continuous enrolment in the health plan prior to randomization<br>-no available telephone contact information	-Medication adherence (PDC)<br><br>-SBP<br><br>-HbA1c<br><br>-LDL	12 months (adherence and HbA1c)<br><br>3 months (SBP)<br><br>7.5 months (LDL)	Intervention	Protocol-structured telephone call using Brief Negotiated Interviewing to ascertain if patient had started to take newly prescribed medication. If so, positive reinforcement; if not filled or not taking, probed for reasons for nonadherence and worked with patient to identify and resolve barriers.<br>The strategies offered to patients by the clinical pharmacists were tailored to activation level and adherence barrier(s) and included: (1) recommendations sent using structured notes with the electronic health record to patients' primary care physicians to modify treatment regimens and coordinate care, (2) follow-up consultations (with the intention that most participants with high activation would receive a maximum of two telephone visits) and (3) strategies to promote adherence including text messages and pillboxes.
							Control	Usual care

Oliveira-Filho 2014<br><br>RT<br><br>Brazil	Established CVD	62	- Patients with discharge diagnosis of CVD<br>-on antihypertensive medication	- If reported already using any tool to improve adherence	-Medication adherence MMAS-8)<br><br>-Hospital readmission	12 months	Intervention	Discharge counseling including habit linking medication with other activity; explaining hypertension and how medication works to manage it and consequences if patient stops taking it; and teaching patient how to monitor common side effects of treatment and support to report any problems to doctor. Also provided with drug treatment schedule as fridge magnet along with phone number for contact.
							Control	Attention control
Persell 2018<br><br>cRT<br><br>USA	Prevention of CVD	920, 12 clusters	-Centres in the access community health network using a common electronic health record.<br>Participants:<br>-age >=18<br>- self-report of 3 of more medications (for any purpose)<br>-SBP 135+ or DBP 85+ at enrolment if no T2DM<br>-/SBP 130+ or DBP 80+ at enrolment if T2DM.<br>-no one else responsible for administering medication<br>- mini Cog exam score of at least 3/5<br>-no intention to change source of care during the next year<br>-ability to communicate in English	-Centres with predominantly non-English speaking patient populations	-Medication adherence (4-day recall)<br><br>-SBP	12 months	Intervention 1: EHR	When patients arrived at health centre for appointment, medication sheet to discuss in consultation was provided, with boxes for patients to tick if concerns about side effects, ability to strike out if not taking, and to write in other meds not included. Also information provided in discussion and written format on purpose and benefits of medication, how to take medication and for how long; when to stop taking and call physician.
							Intervention 2: EHR+ education	1) EHR intervention as above<br>2) Medication counselling by nurse educator; including assessment of comprehension, review of medication use; assistance with regimen dosing consolidation when feasible, development of medication table for complex regimens; assessment of adherence and reasons for non-adherence; addressing misconceptions and reinforced role medications play in disease control using teach back.
							Control	Not described
Schroeder 2005<br><br>RT<br><br>UK	Prevention of CVD	245	-Patients coded as having HTN<br>-latest BP of 150+/90+ in the past 6 months.	-individuals who did not control their medication intake<br>-secondary HTN<br>-severe dementia<br>-'other reasons for not approaching patient' eg bereavement	-Medication adherence (MEMS)<br><br>-SBP	6 months	Intervention	Adherence support session (20 mins), and shorter reinforcement session (10 mins), with nurse, aiming to provide patients with opportunity to talk about any problems with anti-hypertensive drugs in a safe, non-threatening, patient-led atmosphere. Nurses explored whether patients understood their diagnosis and agreed with the treatment process; were encouraged to address patient concerns with medication and to agree tailored strategies to resolve any medication problems

								(such as non-comprehension, forgetfulness), using self-regulatory model of illness behaviour.
							Control	Usual care
Simon 2012<br><br>RT<br><br>India	T2DM	97	-T2DM<br>-Age 18-80<br>-on biguanides, sulfonylureas, or insulin<br>-Hba1C at least 7%	-T1DM<br>-psychiatric illness<br>-pregnancy/lactation<br>-not willing to consent	-Medication adherence (MARS)<br><br>-SBP<br><br>-HbA1c<br><br>-LDL	6 months	Intervention	1 20-minute session of counselling addressing indications of medications, adverse effects, doses/frequencies, medication adherence and reinforcement of lifestyle advice, and patient counselling leaflets as supplementary materials
							Control	Minimal basic counselling regarding medications, diet and follow-up details.
Stamm-Balderjahn 2016<br><br>RT<br><br>Germany	Established CVD	545	-aged between 18-85 with coronary artery disease (acute MI, PCI, or aorto-coronary venous bypass surgery)	-none described	-Medication adherence (MMAS-4)<br><br>-SBP<br><br>-LDL	6 months	Intervention group A: goal setting and checking	1) goal setting interview in which patient and doctor set out and recorded goals for behaviour-related protection and risk factors<br>2) goal checking interview 3 months later with doctor or therapist in which they reflected on goal adherence and offered support if practical implementation caused difficulty.<br>3) provision of info brochure on condition information; and patient passport to document measured values for physiological protection factors and risk factors.
							Intervention group B: goal setting<br><br>Intervention groups pooled for meta-analysis due to similar intervention approach of differing intensity	1) Goal setting interview in which patient and doctor set out and recorded goals for behaviour-related protection factors and risk factors.<br>2) not specifically stated whether patients also received information brochure on disorder specific information and info on risk and protective factors; and patient passport to document measured values for protection and risk factors over time<br>3) no goal checking interview
							Control	Usual care (cardiac rehabilitation)

Sundararajan 2020<br><br>RT<br><br>India	Established CVD	154	-Adults 18+ admitted for MI with PCI or CABG	-Heart transplant, heart valve surgery, heart failure, and other comorbidities	-Medication adherence (MARS)<br><br>-SBP<br><br>-LDL	6 months	Intervention	2 sessions of 30-minute pharmacist counselling 6 months apart, which addressed and documented names of medications, indications, doses/frequencies, adverse effects, medication adherence, physical activity, alcohol limits, and smoking cessation. PILs in local language explained and provided, containing summary of MI symptoms, risk factors, lifestyle modifications, diet counselling, and drug counselling.
							Control	Usual care
Tommelein 2014<br><br>RT<br><br>Belgium	Respiratory disease	734	-Aged >=50<br>-smoking history of at least 10 pack years<br>-prescription for daily COPD maintenance medication<br>-regular visitor to the pharmacy.	-Current asthma<br>-analphabetic	-Medication adherence (MRA)<br><br>-Respiratory symptomatology (COPD assessment test)	3 months	Intervention	2 counselling sessions (each 15-25 mins) with pharmacist; structured patient education on COPD pathophysiology, medication, technique, importance of adherence, lifestyle advice. Depending on reason for non-adherence pharmacist could advise reminders, discuss habit linking, address concerns
							Control	Usual pharmacist care
Van der Laan 2019<br><br>RT<br><br>The Netherlands	Prevention of CVD	170	-Patients 45-75<br>-using antihypertensive medication for at least 12 months<br>-with hypertension by self-report<br>-Non-adherence according to dispensing data: PDC <80% for at least one antihypertensive drug class in last 6 months, and MARS5 <25	-insufficient Dutch language<br>-used medication support services from the pharmacy eg pill packaging	-Medication adherence (MARS5)<br><br>-SBP	9 months (adherence)<br><br>6 months (SBP)	Intervention	1) Pharmacists undertook interview (average 36 mins) to explore barriers to medication adherence and discussed one or more of: information about medications, dealing with side effects, overcoming practical problems; diminishing negative beliefs.<br>2) Follow-up consultation (average 20 mins) after 3 months to discuss patients' implementation of experiences with discussed info and recommendations
							Control	Usual care
Ting 2021<br><br>RT<br><br>Malaysia	T2DM	131	- Malay T2DM Patients<br>-HbA1c level greater than 7%<br>- SEAMS total score	- pregnant women<br>-patients less than 18 years old<br>- severe and enduring mental health problems	-Medication adherence (SEAMS)<br><br>-HbA1c	12 months	Intervention	1 group education session for 20-24 patients, delivered by 4 facilitator over 3 hours,, including 'mythbusters', 'World Café' and 'role play' approaches to address intentional non-adherence. Participants shared difficulties in

			less than 26 upon recruitment	-unable to listen or read due to inherited disabilities or other issues<br>-unable to communicate in the Malay language<br>-participating in other studies<br>-declining consent to participate<br>-hospitalised patients.				adhering to medications with other group members and brainstormed solutions that could work for each of them. Services provided by health clinics that address difficulties in collecting medication supplies also signposted.
							Control	Usual care
**Unclear**								
Carter 2009<br><br>cRT<br><br>USA	Prevention of CVD	402, 6 clusters	-aged 21 and older<br>-taking 0 to 3 antihypertensives<br>- if no diabetes: SBP between 140 and 179, DBP between 90 and 109<br>- if diabetes: SBP 130 to 179, DBP 80 to 109	- cognitive impairment<br>-pregnancy<br>-unstable angina<br>-serious renal or hepatic disease<br>-BP of 180/110mmHg or higher<br>-poor prognosis (life expectancy 3 years)<br>-evidence of hypertensive urgency or emergency<br>-New York Heart Association class III or IV heart failure, myocardial infarction or stroke<br>-antihypertensive medication or dosage change within 4 weeks of the baseline visit	-Medication adherence (MMAS-4)<br><br>-SBP	6 months	Intervention	1) Physician and pharmacists undertook team building exercises.<br>2) Pharmacists encouraged to assess medications at 1 and 3 months, and provide recommendations consistent with national guidelines; physicians and pharmacists decided how best to implement the intervention and were not required to perform the suggested intervention visits. (Mean 1.6 SD 1.4 contacts per patient)
							Control	Usual care
Pladevall 2015<br><br>RT<br><br>USA	Multiple	569	-Age 18 or over<br>-at least 1 of: Hba1c measurement with the last value >=7%, LDL-C measurement with the last value >=100mg/dL<br>-prescription for both an oral diabetes med and a lipid-lowering medication<br>-member of a health plan with prescription drug coverage in 2007 and 2008	-hospice care<br>-hospitalized for over 90 days;<br>-primary care provider didn't consent	-Medication adherence (PDC)<br><br>-HbA1c<br><br>-LDL	12 months	Intervention group 1: adherence information	Measure of medication adherence (prescription fill information) provided as automated electronic message to primary care physician via electronic prescription writing application. Clinicians could view these data (along with blood results, and trends in adherence over time) at time of reviewing, writing, and refilling prescriptions. Were given instruction on how to interpret and discuss these with their patients.
							Intervention group 2: adherence information + motivational interviewing	1) Provision of electronic information on medication adherence to primary care physicians (as in intervention group 1).<br>2) Motivational interviewing sessions up to every 3 months (mean session number 1.15)
							Control	Minimal training: all physicians given limited training in how to intervene on poor adherence

Risk of Bias

Risk of bias assessment was carried out using Cochrane Risk of Bias 2 tool; data visualization using robvis tool (7)

Traffic light plot of included Parallel Randomised Trials:

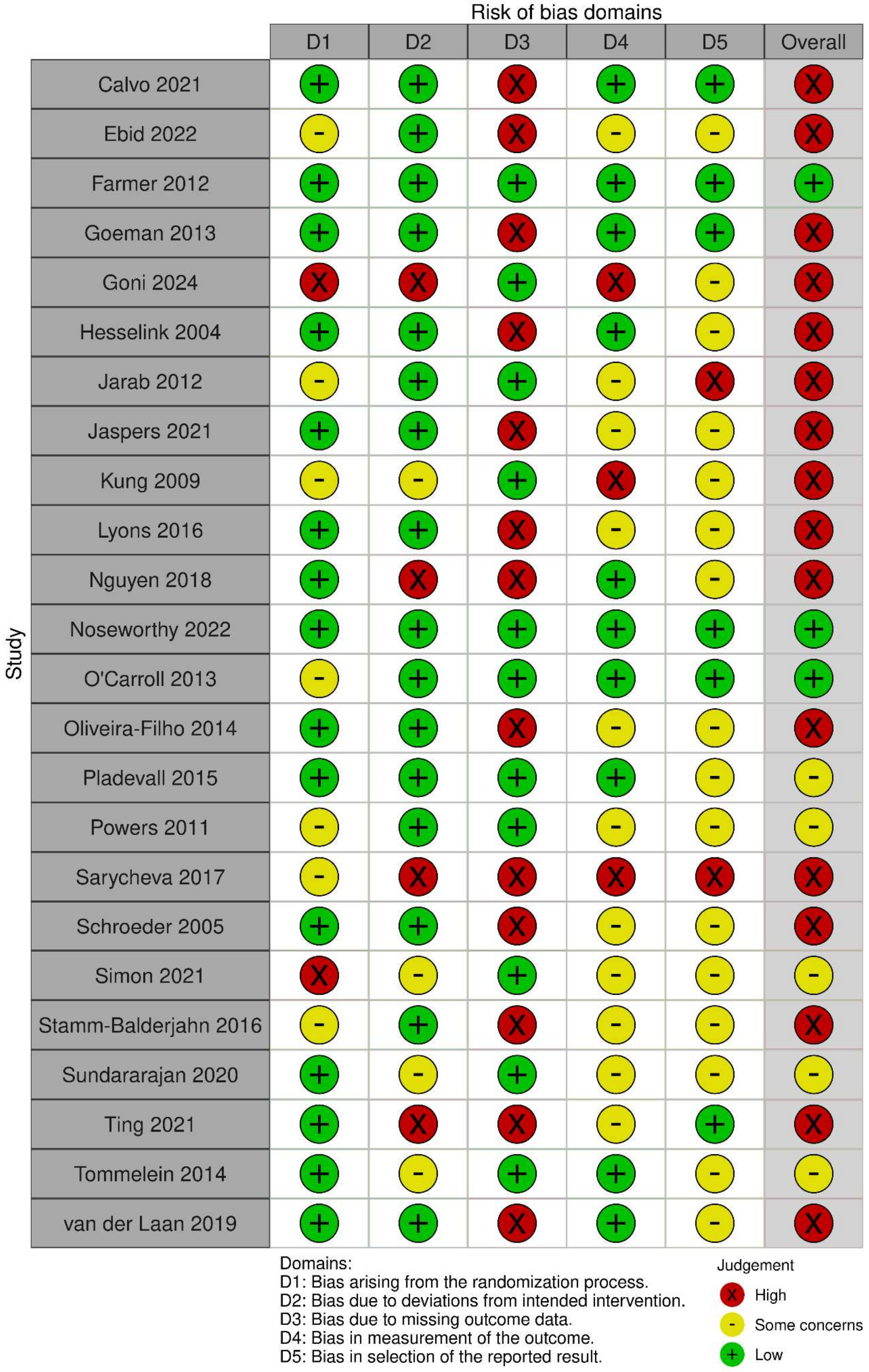

Traffic light plot of included Cluster-Randomised Trials:

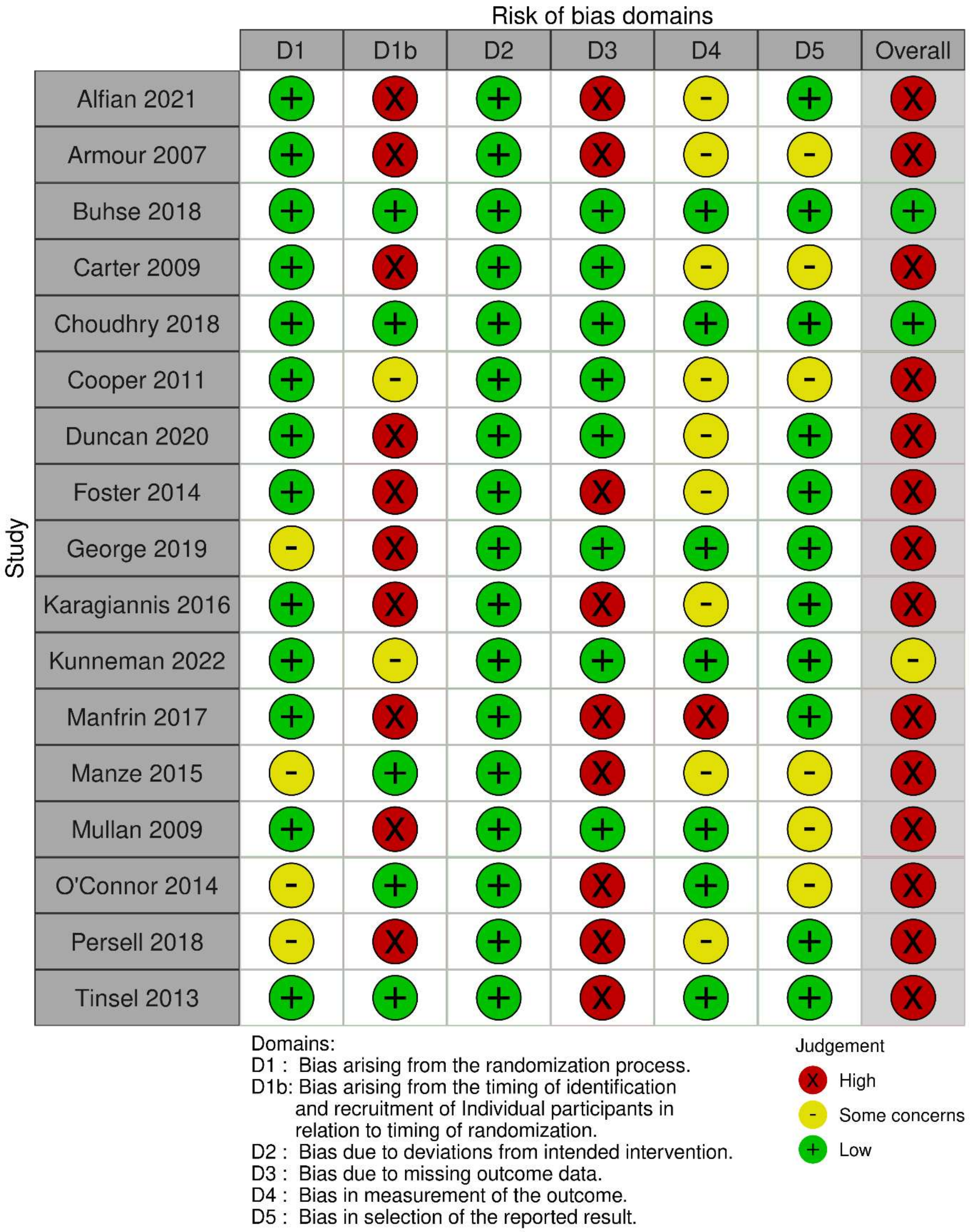

Supplemental Table 5: GRADE assessment of certainty of evidence

Outcome	Certainty of the Evidence Domains (Quality of Evidence)					Certainty (Quality) of the Evidence
	Risk of Bias	Inconsistency	Indirectness	Imprecision	Publication bias	
Medication adherence: primary prevention CVD	Very serious	Not serious	Not serious	Not serious	Undetected	Low[1]
Medication adherence: secondary prevention CVD	Serious	Serious	Not serious	Serious	Strongly suspected	Very low[2]
Medication adherence: T2DM	Very serious	Serious	Not serious	Serious	Strongly suspected	Very low[3]
Medication adherence: Respiratory disease	Very serious	Not serious	Not serious	Not serious	Insufficient studies to assess	Low[4]
Medication adherence: osteoporosis	Very serious	Not serious	Not serious	Serious	Insufficient studies to assess	Very low[5]
Medication adherence: Multiple diseases	Serious	Serious	Not serious	Serious	Insufficient studies to assess	Very low[6]

SBP	Serious	Not serious	Not serious	Not serious	Undetected	Moderate[7]
HbA1c	Serious	Serious	Not serious	Serious	Strongly suspected	Very low[8]
LDL	Serious	Serious	Not serious	Not serious	Undetected	Low[9]
Respiratory symptoms	Very serious	Not serious	Not serious	Not serious	Insufficient studies to assess	Low[10]
Osteoporosis symptoms	Very serious	Not serious	Not serious	Serious	Insufficient studies to assess	Very low[11]
Rehospitalization	Serious	Not serious	Not serious	Serious	Insufficient studies to assess	Low[12]

GRADE Working Group grade of evidence (8).

Moderate certainty: we are moderately confident in the effect estimate: The true effect is likely to be close to the estimate of the effect, but there is a possibility that it is substantially different.

Low certainty: our confidence in the effect estimate is limited: the true effect may be substantially different from the estimate of the effect.

Very low certainty: we have very little confidence in the effect estimate: the true effect is likely to be substantially different from the estimate of effect.

[1] Risk of bias: downgraded by 2 levels due to most studies being at high risk of bias, with critical limitations for 2 criteria.

[2] Risk of bias: downgraded by 1 level due to most studies being at high risk of bias, with critical limitation in 1 criteria. Inconsistency: downgraded by 1 level due to high statistical heterogeneity. Imprecision: downgraded by 1 level due to wide confidence intervals which encompass both clinically significant benefit and clinically significant harm. Publication bias: downgraded by 1 level due to numerous studies with outlying positive estimates of effect.

[3] Risk of bias: downgraded by 2 levels due to most studies being at high risk of bias, with critical limitations for 2 criteria Inconsistency: downgraded by 1 level due to high statistical heterogeneity. Imprecision: downgraded by 1 level due to wide confidence intervals which encompass both clinically significant as well as trivial clinical benefit.

[4] Risk of bias: downgraded by 2 levels due to most studies being at high risk of bias, with critical limitations for 2 criteria.

[5] Risk of bias: downgraded by 2 levels as the sole study included was at high risk of bias, with critical limitation in 1 criterion and some limitations for multiple other criteria. Imprecision: downgraded by 1 level due to wide CIs encompassing both clinically significant benefit and clinically significant harm.

[6] Risk of bias: downgraded by 1 level due to most studies being at high risk of bias, with critical limitation in 1 criterion. Inconsistency: downgraded by 1 level due to high statistical heterogeneity. Imprecision: downgraded by 1 level due to CIs encompassing clinically significant benefit as well as no effect.

[7] Risk of bias: downgraded by 1 level due to most studies being at high risk of bias, with critical limitation in 1 criterion.

[8] Risk of bias: downgraded by 1 level due to most studies being at high risk of bias, with critical limitation in 1 criterion. Inconsistency: downgraded by 1 level due to high statistical heterogeneity. Imprecision: downgraded by 1 level due to wide CIs wide encompassing clinically significant benefit as well as trivial clinical benefit. Publication bias: Downgraded by 1 level because funnel plot was strongly suggestive of publication bias.

[9] Risk of bias: downgraded by 1 level due to most studies being at high risk of bias, with critical limitation in 1 criterion. Inconsistency: downgraded by 1 level due to high statistical heterogeneity.

[10] Risk of bias: downgraded by 2 levels due to most studies being at high risk of bias, with critical limitations for 2 criteria.

[11] Risk of bias: downgraded by 2 levels as the sole study included was at high risk of bias, with critical limitation in 1 criterion and some limitations for multiple other criteria. Imprecision: downgraded by 1 level due to small sample size and no Cis reported.

[12] Risk of bias: downgraded by 1 level due to most studies being at high risk of bias, with critical limitation in 1 criterion. Imprecision: downgraded by 1 level due to wide CIs which encompass clinically-significant harm as well as clinically-significant benefit.

Supplemental Figures

Supplemental Figure 1: Standardised mean difference in adherence, sensitivity analysis using HKSJ model

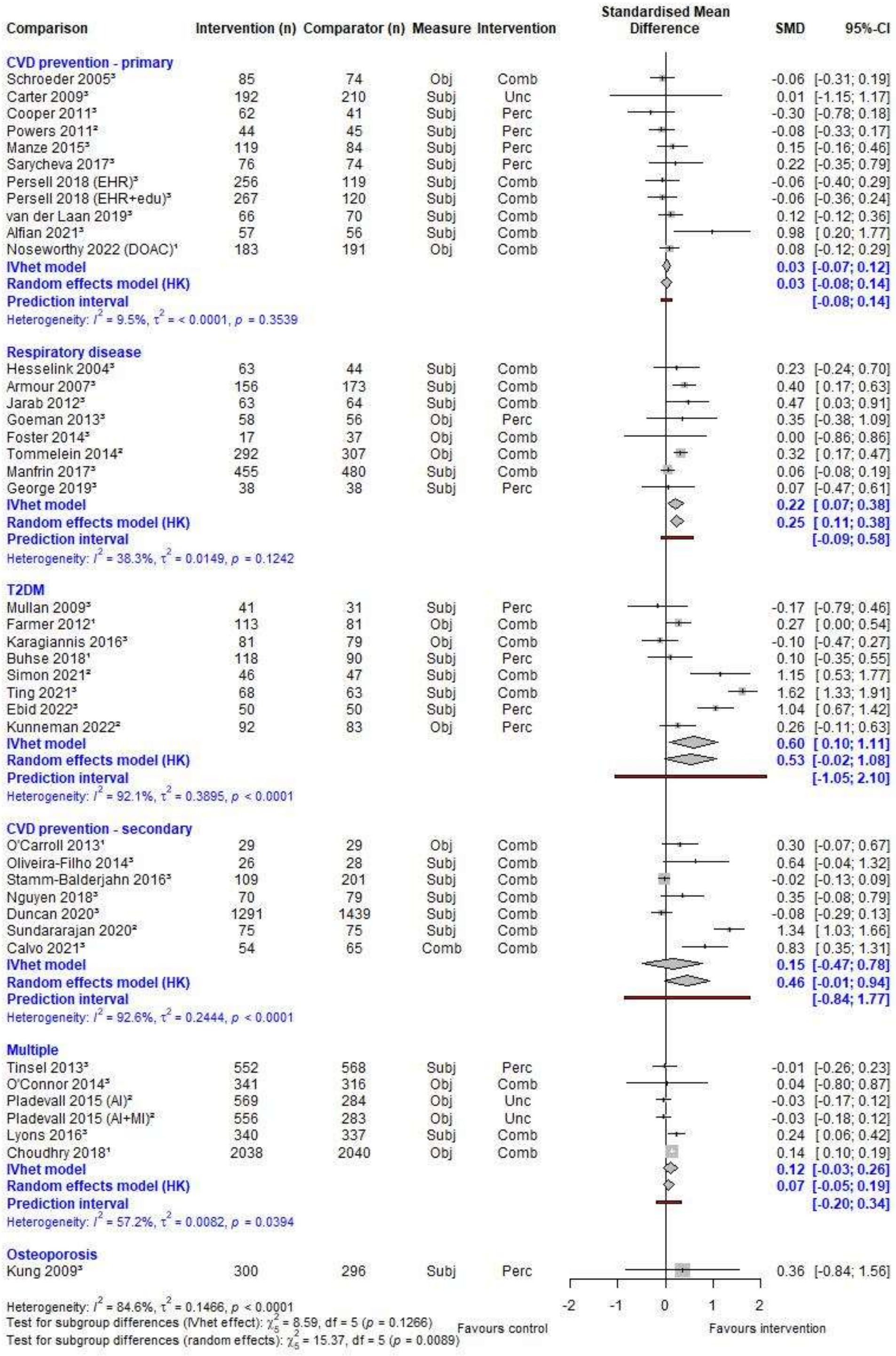

Supplemental Figure 2: Standardised mean difference in adherence, excluding studies at high risk of bias

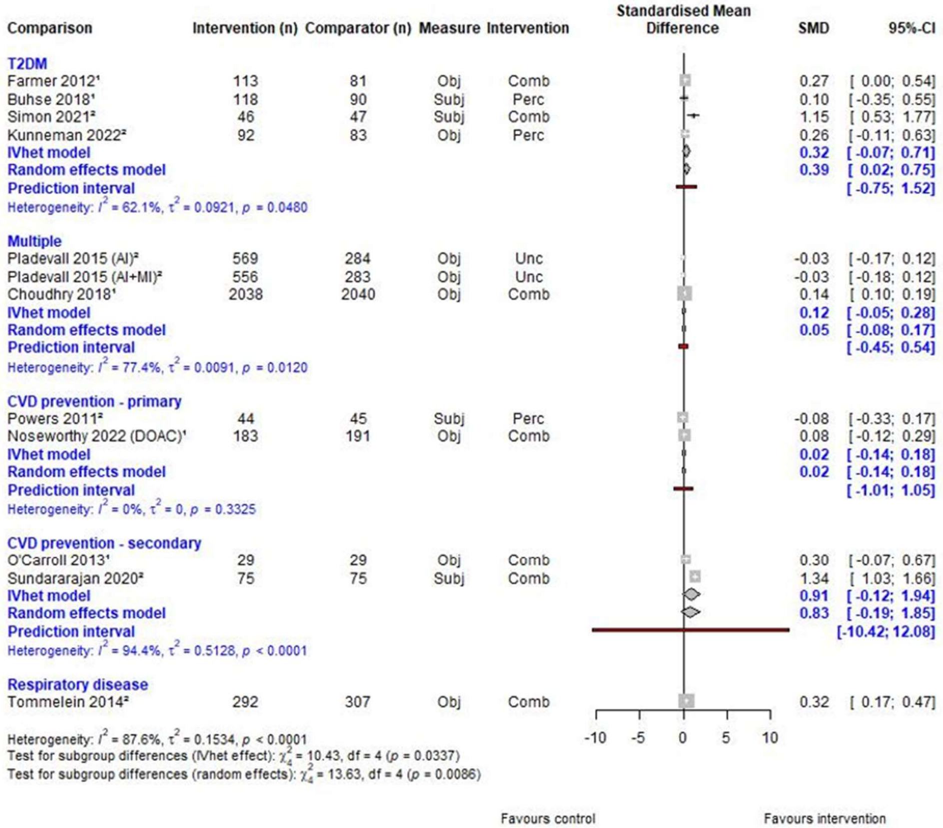

Supplemental Figure 3: Standardised mean difference in adherence, limited to studies in which adherence was poor at baseline

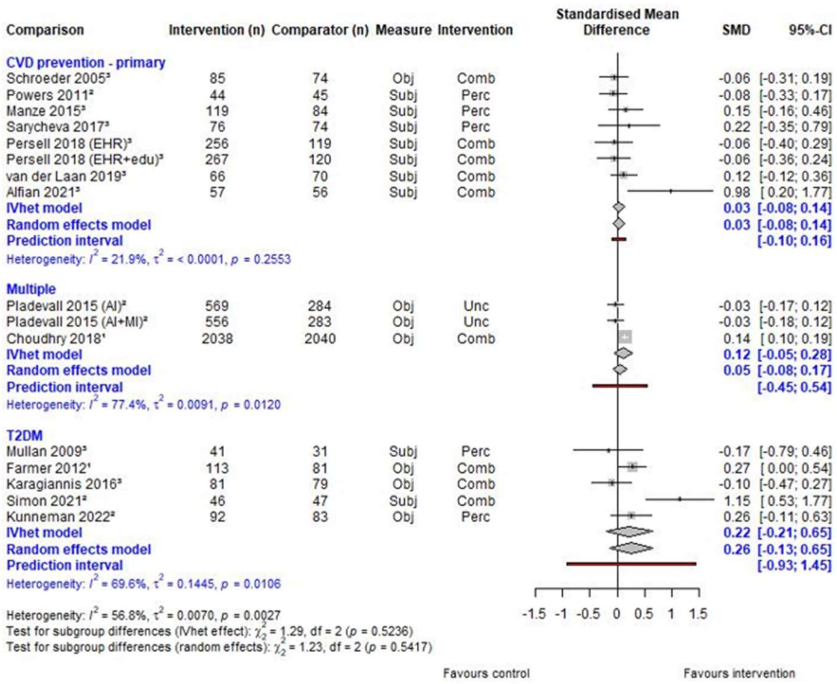

Supplemental Figure 4: standardised mean difference in medication adherence, subgroup analysis by type of intervention

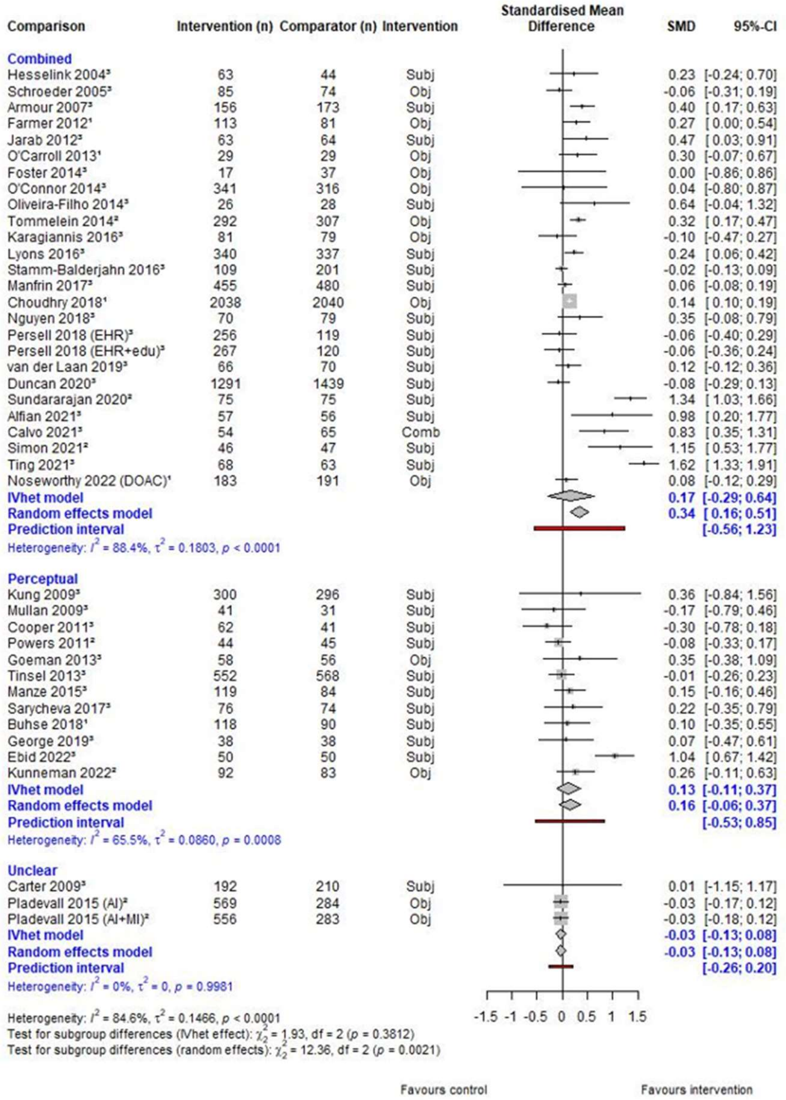

Supplemental Figure 5: standardised mean difference in medication adherence, subgroup analysis by number of patient contacts required by intervention

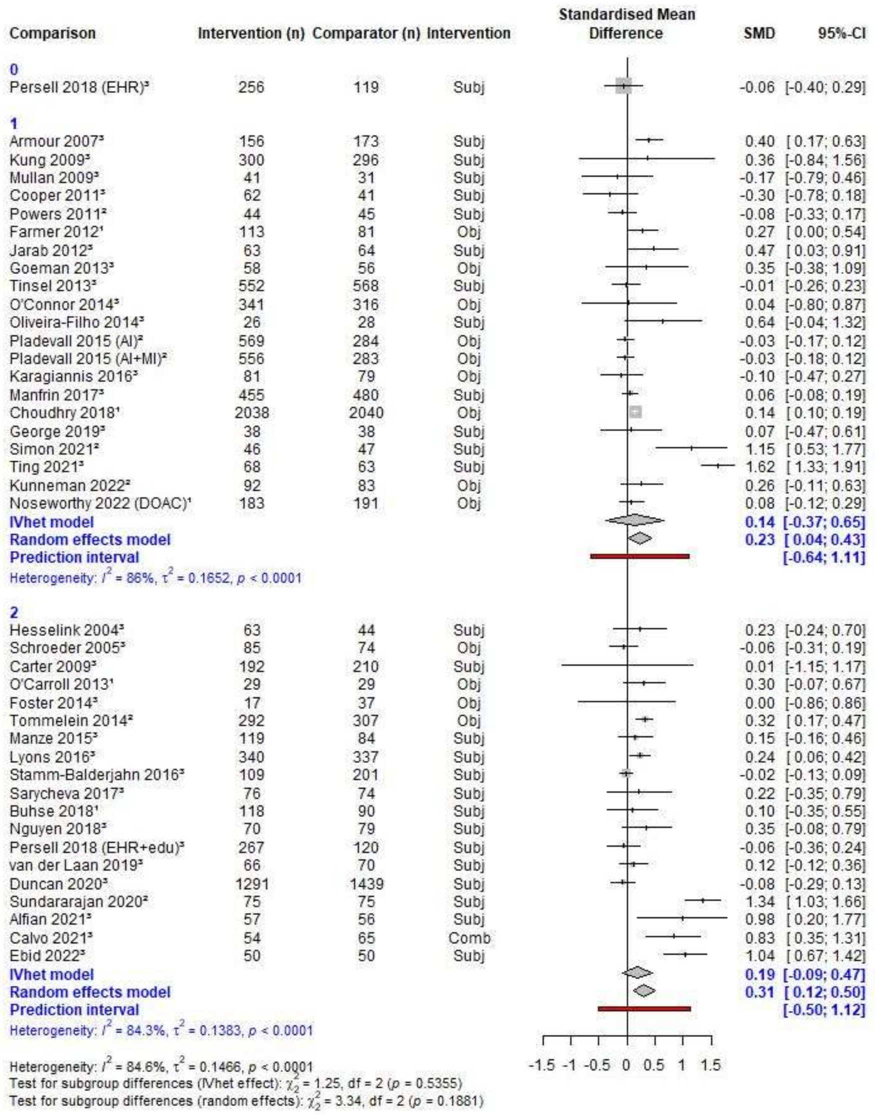

Supplemental Figure 6: funnel plot for primary adherence outcome, studies colour-coded by disease group

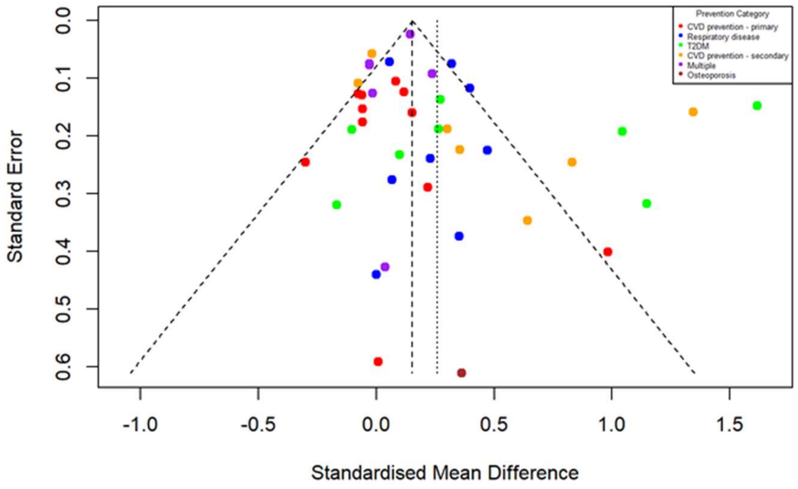

Supplemental Figure 7: Mean difference in SBP, primary analysis using IVHet model

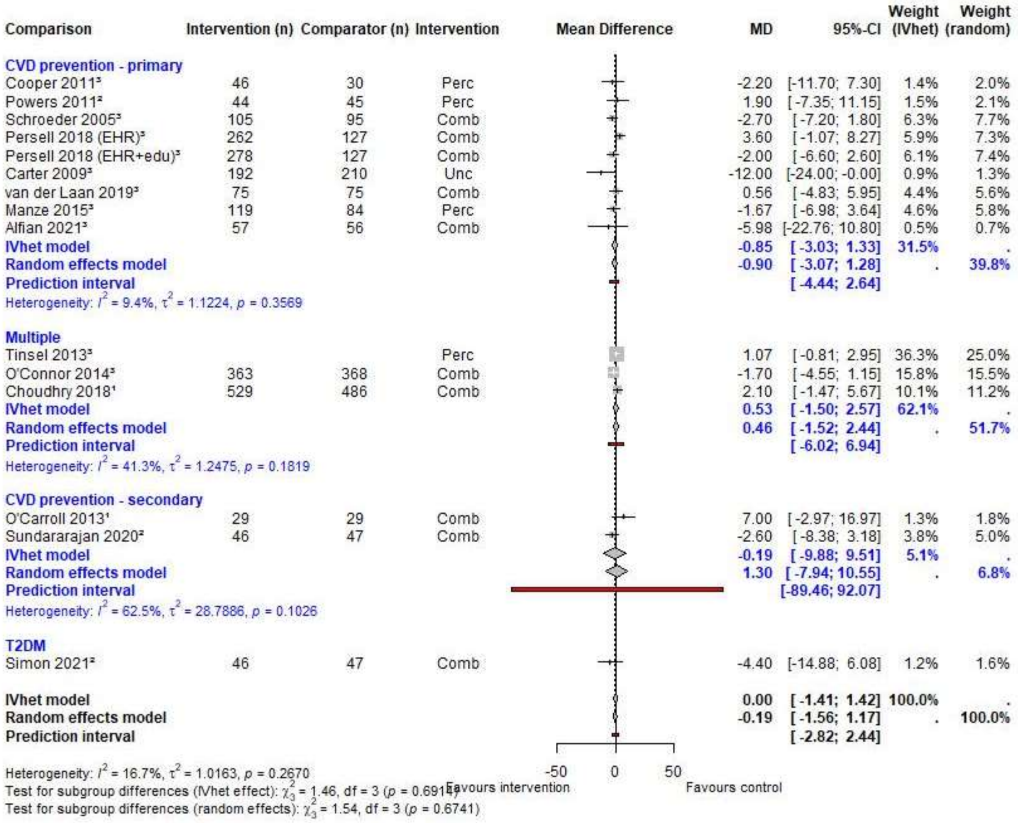

Two studies could not be included in meta-analysis; one of which found no significant effect of the intervention on change in blood pressure (9), and one finding significantly lower blood pressure at endpoint in the intervention group (10).

Supplemental Figure 8: Mean difference in SBP, sensitivity analysis using HKSJ model

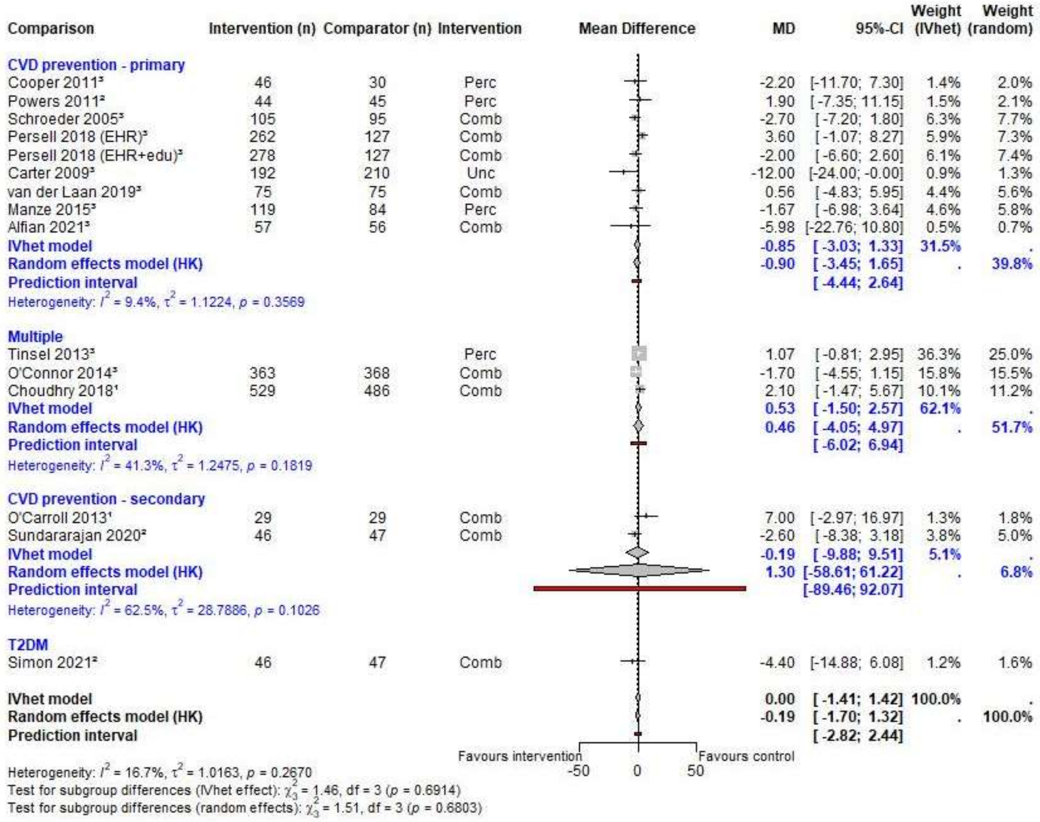

Supplemental Figure 9: Mean difference in SBP, excluding studies at high risk of bias

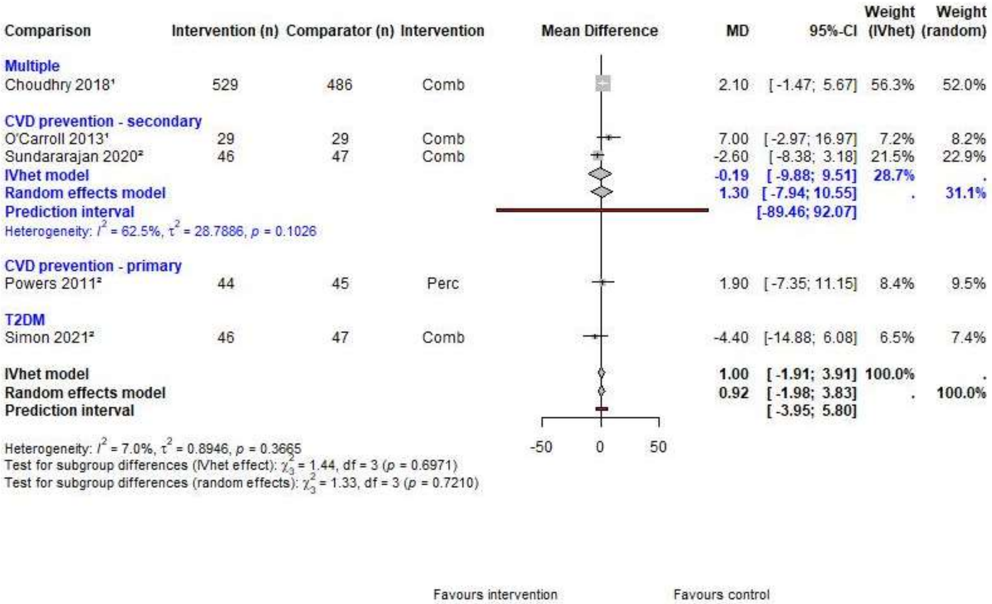

Supplemental Figure 10: Mean difference in SBP, limited to studies in which SBP was poorly controlled (as defined by study authors) at baseline

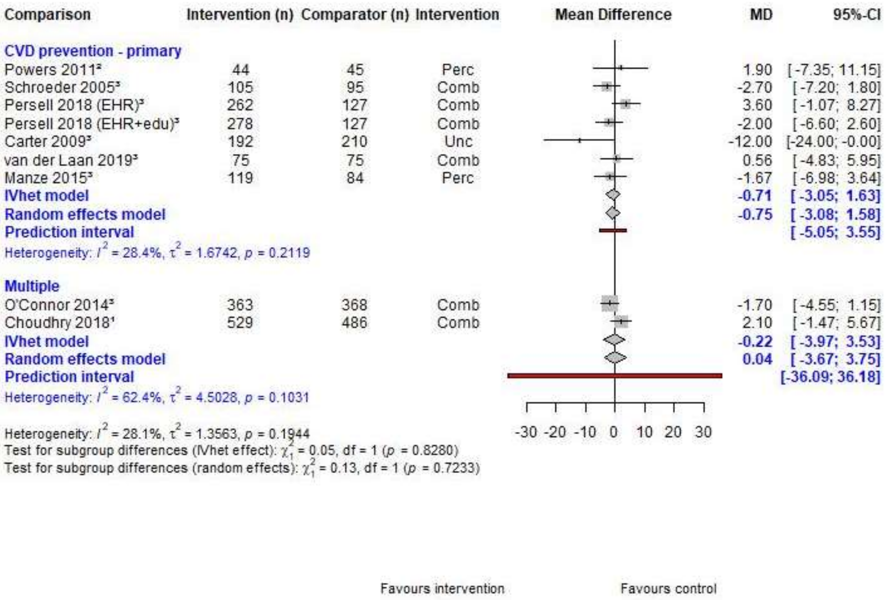

Supplemental Figure 11: funnel plot for SBP outcome

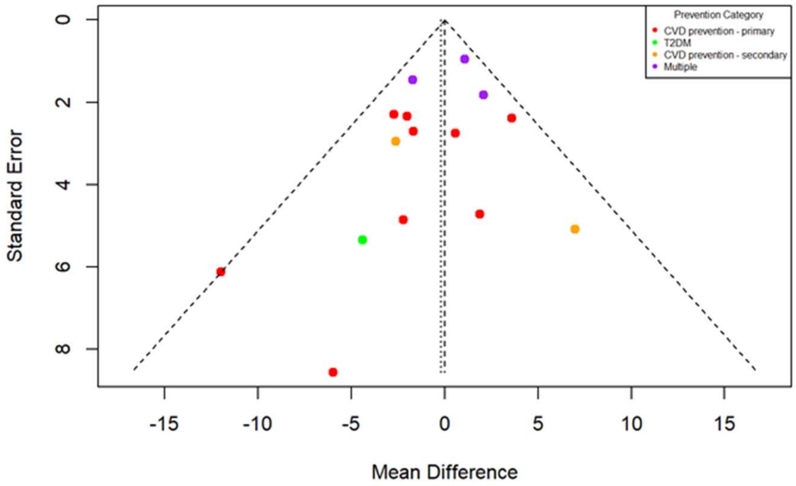

Supplemental Figure 12: Mean difference in HbA1c, primary analysis using IVHet model

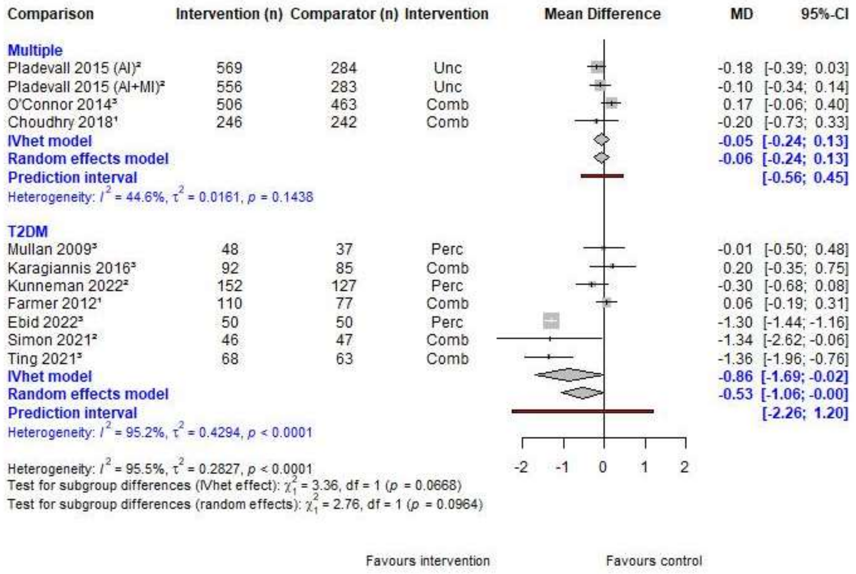

Two studies assessing this outcome could not included in meta-analysis (neither finding significant difference in odds of achieving goal HbA1c (11,12)).

Supplemental Figure 13: Mean difference in HbA1c, sensitivity analysis using HKSJ model

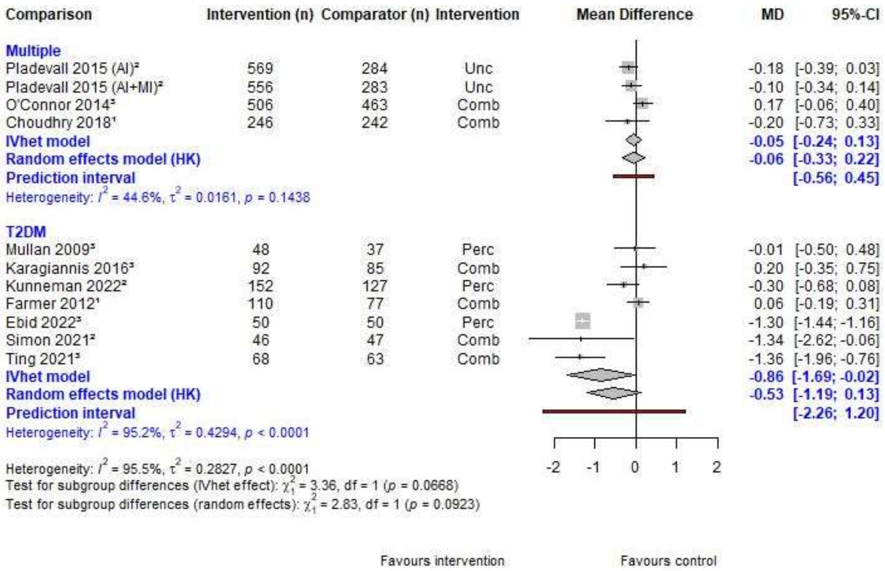

Supplemental Figure 14: Mean difference in HbA1c, excluding studies at high risk of bias

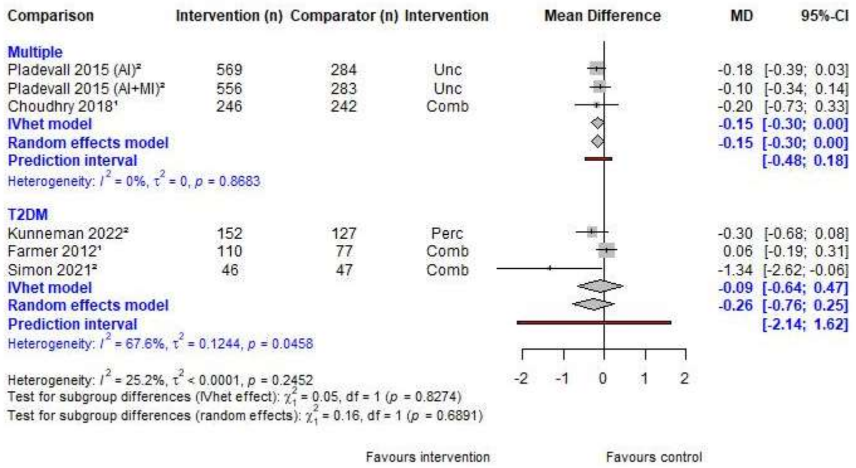

Supplemental Figure 15: Mean difference in HbA1c, limited to studies in which HbA1c was poorly controlled (as defined by study authors) at baseline

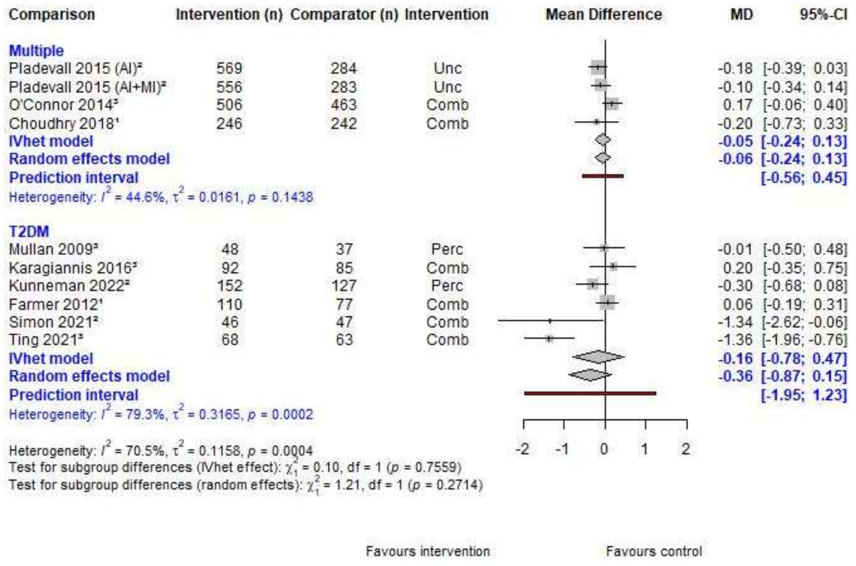

Supplemental Figure 16: funnel plot for HbA1c outcome

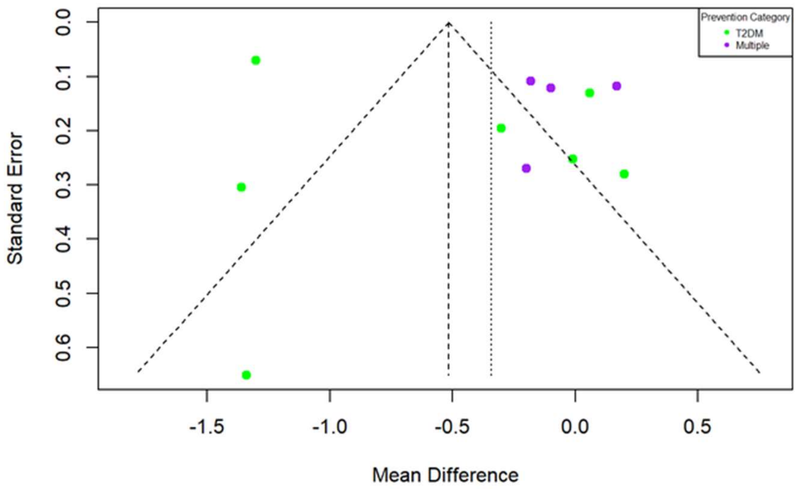

Supplemental Figure 17: post-hoc sensitivity analysis for HbA1c outcome

Excluding one outlier study with 100 participants and unusually low reported variance (4)

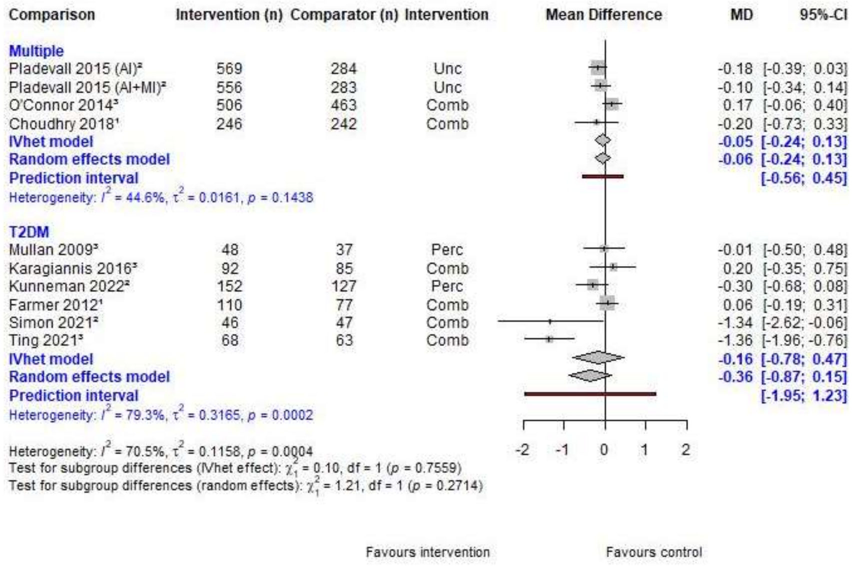

Supplemental Figure 18: Mean difference in LDL, primary analysis using IVHet model

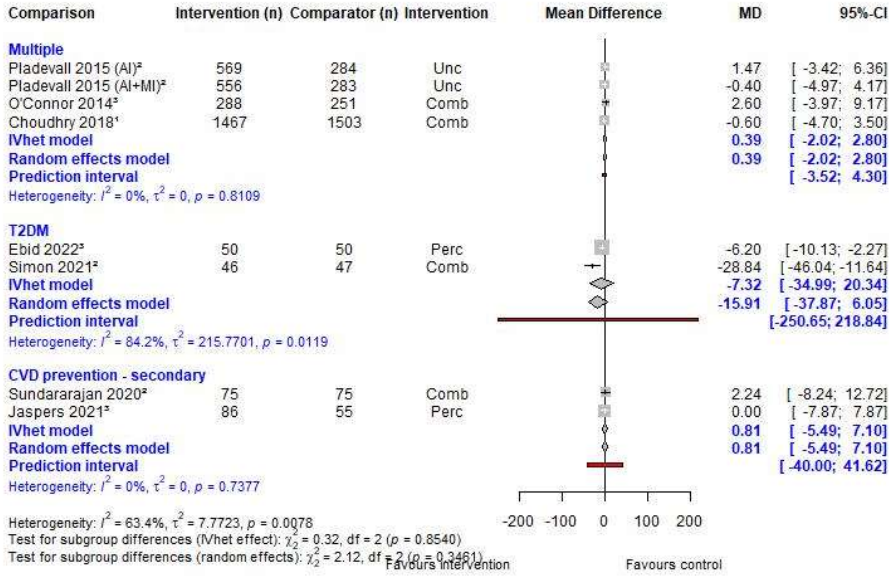

Two studies could not be included in meta-analysis, with one finding no evidence of effect of the intervention on LDL (9), and one finding significantly lower LDL at endpoint in the intervention group (10).

Supplemental Figure 19: Mean difference in LDL, sensitivity analysis using HKSJ meta-analysis method

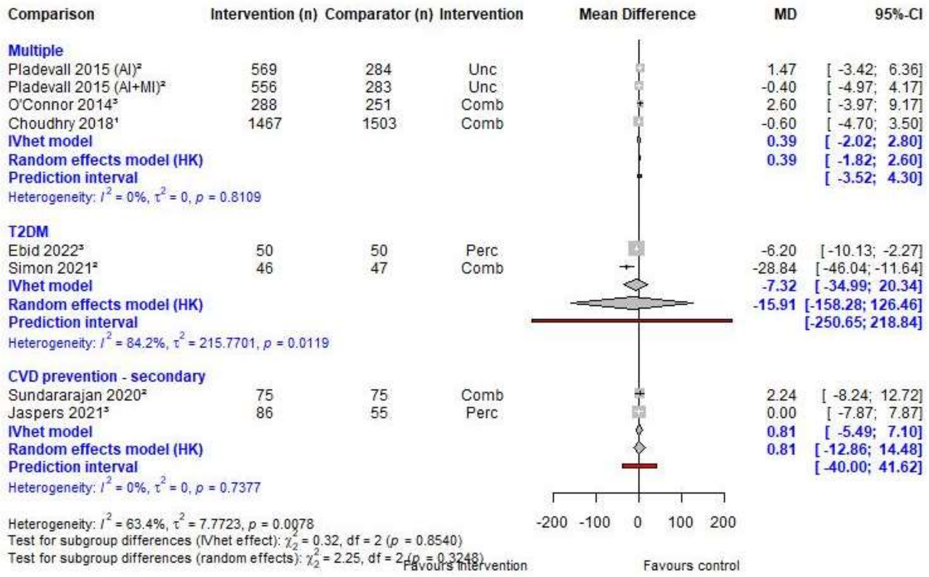

Supplemental Figure 20: Mean difference in LDL, excluding studies at high risk of bias

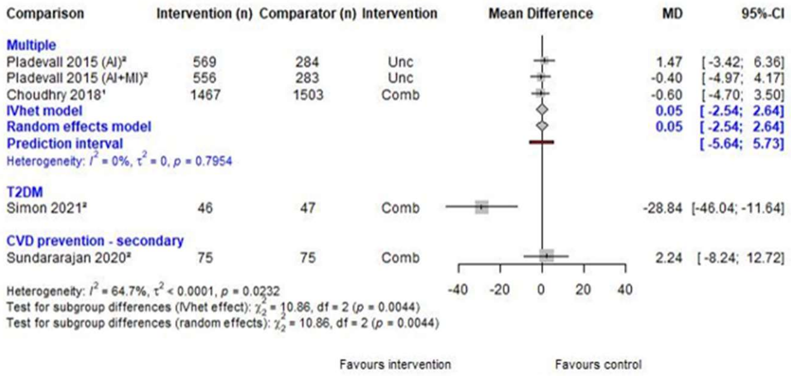

Supplemental Figure 21: Mean difference in LDL, limited to studies in which LDL was poorly controlled (as defined by study authors) at baseline

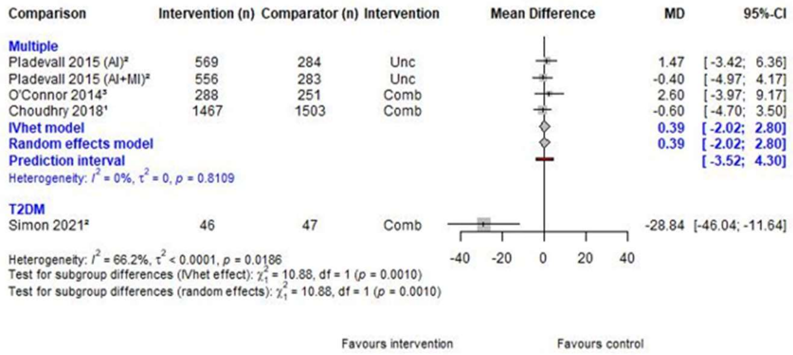

Supplemental Figure 22: funnel plot for LDL outcome

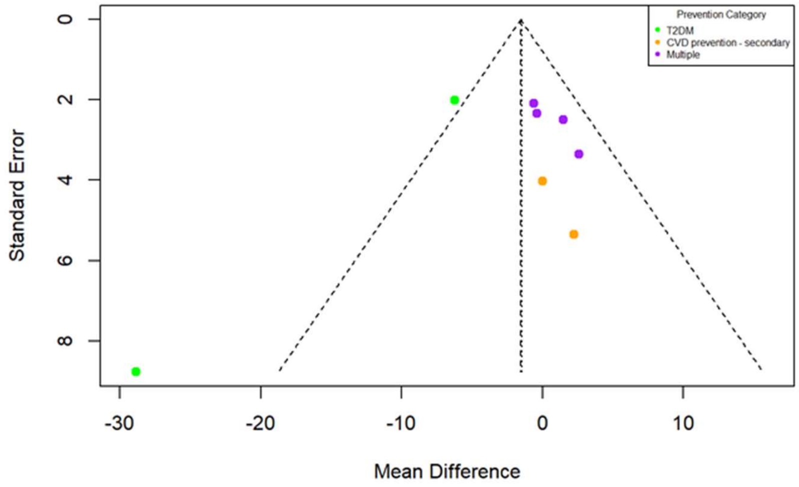

Supplemental figure 23: Odds ratio of hospitalization, primary analysis using IVHet model

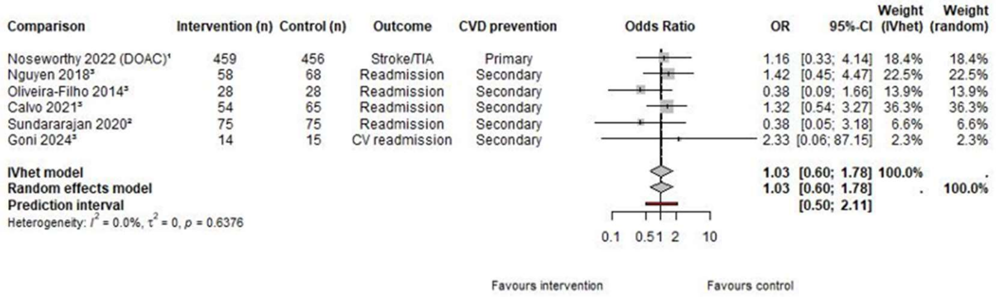

Supplemental figure 24: Odds ratio of hospitalization, sensitivity analysis using HKSJ model

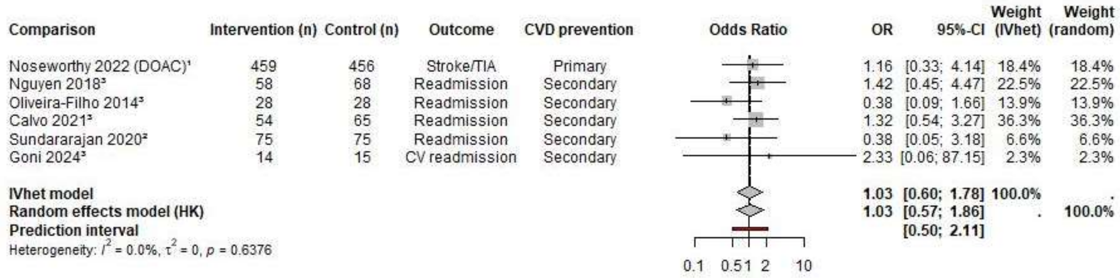

Supplemental Figure 25: Standardised mean difference in respiratory symptoms, primary analysis using IVHet model

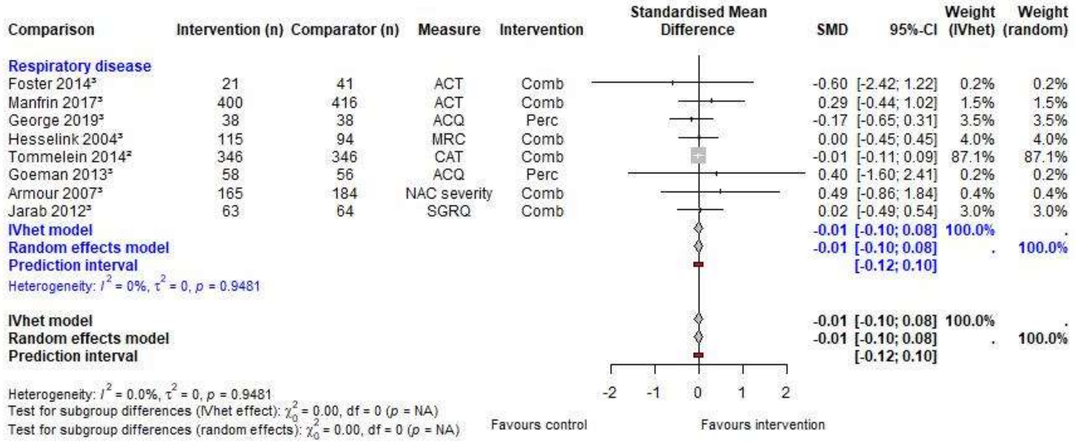

Measures of respiratory disease symptoms included asthma control (ACT: Asthma Control Test; ACQ: Asthma Control Questionnaire), asthma severity (NAC severity: National Asthma Council of Australia's asthma severity assessment table), COPD control (CAT: COPD assessment test) and respiratory symptoms (MRC: Medical Research Council dyspnoea scale; SGRQ: St George's Respiratory Questionnaire).

Supplemental Figure 26: Standardised mean difference in respiratory symptoms, sensitivity analysis using HKSJ meta-analysis method

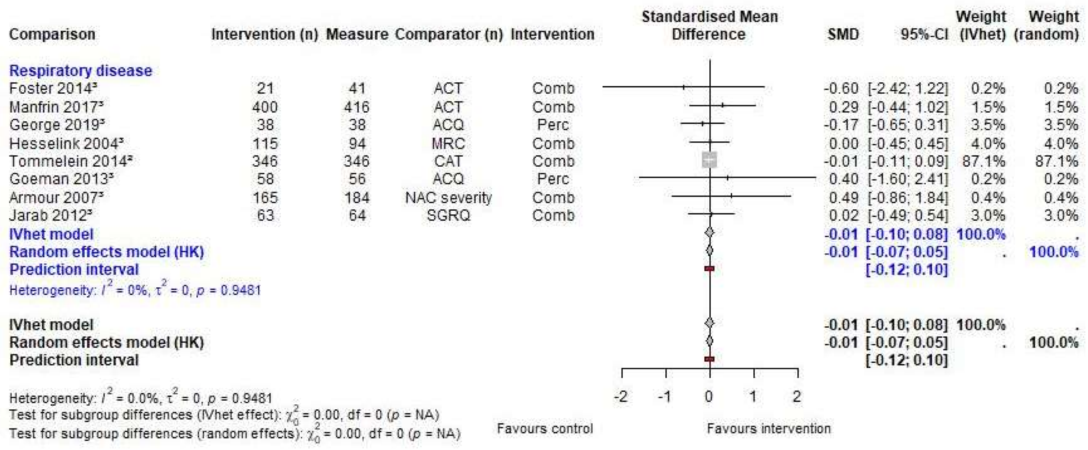

Supplemental Figure 27: Standardised mean difference in respiratory symptoms, limited to studies in which respiratory symptoms were poorly controlled (as defined by study authors) at baseline

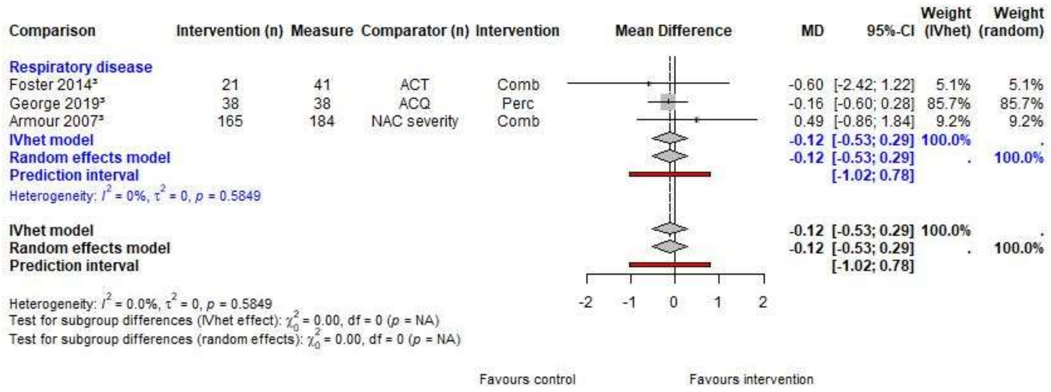

Supplemental Figure 28: funnel plot for respiratory symptom outcome

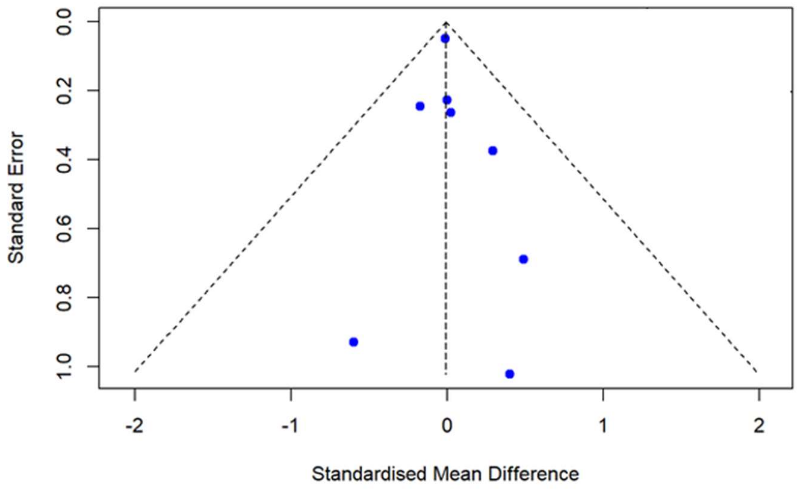

PRISMA 2020 Checklist

Section and Topic	Item #	Checklist item	Location where item is reported
**TITLE**			
Title	1	Identify the report as a systematic review.	Title
**ABSTRACT**			
Abstract	2	See the PRISMA 2020 for Abstracts checklist.	Abstract
**INTRODUCTION**			
Rationale	3	Describe the rationale for the review in the context of existing knowledge.	Background
Objectives	4	Provide an explicit statement of the objective(s) or question(s) the review addresses.	Objective
**METHODS**			
Eligibility criteria	5	Specify the inclusion and exclusion criteria for the review and how studies were grouped for the syntheses.	Eligibility criteria; table 1; tables S1-3
Information sources	6	Specify all databases, registers, websites, organisations, reference lists and other sources searched or consulted to identify studies. Specify the date when each source was last searched or consulted.	Data sources and searches
Search strategy	7	Present the full search strategies for all databases, registers and websites, including any filters and limits used.	Supp material
Selection process	8	Specify the methods used to decide whether a study met the inclusion criteria of the review, including how many reviewers screened each record and each report retrieved, whether they worked independently, and if applicable, details of automation tools used in the process.	Screening
Data collection process	9	Specify the methods used to collect data from reports, including how many reviewers collected data from each report, whether they worked independently, any processes for obtaining or confirming data from study investigators, and if applicable, details of automation tools used in the process.	Screening
Data items	10a	List and define all outcomes for which data were sought. Specify whether all results that were compatible with each outcome domain in each study were sought (e.g. for all measures, time points, analyses), and if not, the methods used to decide which results to collect.	Data synthesis and analysis; supp materials
	10b	List and define all other variables for which data were sought (e.g. participant and intervention characteristics, funding sources). Describe any assumptions made about any missing or unclear information.	Supp materials
Study risk of bias assessment	11	Specify the methods used to assess risk of bias in the included studies, including details of the tool(s) used, how many reviewers assessed each study and whether they worked independently, and if applicable, details of automation tools used in the process.	Screening
Effect measures	12	Specify for each outcome the effect measure(s) (e.g. risk ratio, mean difference) used in the synthesis or presentation of results.	Data synthesis and analysis
Synthesis methods	13a	Describe the processes used to decide which studies were eligible for each synthesis (e.g. tabulating the study intervention characteristics and comparing against the planned groups for each synthesis (item #5)).	Data synthesis and analysis; supp materials

	13b	Describe any methods required to prepare the data for presentation or synthesis, such as handling of missing summary statistics, or data conversions.	Supp materials
	13c	Describe any methods used to tabulate or visually display results of individual studies and syntheses.	
	13d	Describe any methods used to synthesize results and provide a rationale for the choice(s). If meta-analysis was performed, describe the model(s), method(s) to identify the presence and extent of statistical heterogeneity, and software package(s) used.	Data synthesis and analyses
	13e	Describe any methods used to explore possible causes of heterogeneity among study results (e.g. subgroup analysis, meta-regression).	Data synthesis and analysis
	13f	Describe any sensitivity analyses conducted to assess robustness of the synthesized results.	Data synthesis and analysis
Reporting bias assessment	14	Describe any methods used to assess risk of bias due to missing results in a synthesis (arising from reporting biases).	Data synthesis and analysis
Certainty assessment	15	Describe any methods used to assess certainty (or confidence) in the body of evidence for an outcome.	Data synthesis and analysis
**RESULTS**			
Study selection	16a	Describe the results of the search and selection process, from the number of records identified in the search to the number of studies included in the review, ideally using a flow diagram.	Figure 1
	16b	Cite studies that might appear to meet the inclusion criteria, but which were excluded, and explain why they were excluded.	N/A
Study characteristics	17	Cite each included study and present its characteristics.	Supp table 4
Risk of bias in studies	18	Present assessments of risk of bias for each included study.	Supp materials
Results of individual studies	19	For all outcomes, present, for each study: (a) summary statistics for each group (where appropriate) and (b) an effect estimate and its precision (e.g. confidence/credible interval), ideally using structured tables or plots.	Figure 2; Supplemental figures
Results of syntheses	20a	For each synthesis, briefly summarise the characteristics and risk of bias among contributing studies.	Supp materials
	20b	Present results of all statistical syntheses conducted. If meta-analysis was done, present for each the summary estimate and its precision (e.g. confidence/credible interval) and measures of statistical heterogeneity. If comparing groups, describe the direction of the effect.	Table 2
	20c	Present results of all investigations of possible causes of heterogeneity among study results.	Supp figures
	20d	Present results of all sensitivity analyses conducted to assess the robustness of the synthesized results.	Supp figures
Reporting biases	21	Present assessments of risk of bias due to missing results (arising from reporting biases) for each synthesis assessed.	Supp figures
Certainty of evidence	22	Present assessments of certainty (or confidence) in the body of evidence for each outcome assessed.	Supp talbe 5
**DISCUSSION**			
Discussion	23a	Provide a general interpretation of the results in the context of other evidence.	Comparison with existing literature
	23b	Discuss any limitations of the evidence included in the review.	Strengths and limitations
	23c	Discuss any limitations of the review processes used.	Strengths and limitations
	23d	Discuss implications of the results for practice, policy, and future research.	Future implications
**OTHER INFORMATION**			

Registration and protocol	24a	Provide registration information for the review, including register name and registration number, or state that the review was not registered.	Method and references
	24b	Indicate where the review protocol can be accessed, or state that a protocol was not prepared.	References
	24c	Describe and explain any amendments to information provided at registration or in the protocol.	Protocol
Support	25	Describe sources of financial or non-financial support for the review, and the role of the funders or sponsors in the review.	Funding statement
Competing interests	26	Declare any competing interests of review authors.	Competing interests statement
Availability of data, code and other materials	27	Report which of the following are publicly available and where they can be found: template data collection forms; data extracted from included studies; data used for all analyses; analytic code; any other materials used in the review.	Data availability statement

References

1. Nieuwlaat R, Wilczynski N, Navarro T, Hobson N, Jeffery R, Keepanasseril A, et al. Interventions for enhancing medication adherence. Cochrane Database of Systematic Reviews [Internet]. 2014;(11). Available from: https://www.cochranelibrary.com/cdsr/doi/10.1002/14651858.CD000011.pub4/full
2. Conn VS, Ruppar TM. Medication adherence outcomes of 771 intervention trials: Systematic review and meta-analysis. Prev Med (Baltim) [Internet]. 2017 Jun;99:269–76. Available from: https://www.sciencedirect.com/science/article/pii/S0091743517301044
3. Multidisciplinary Team Working in a General Practice Setting.
4. Ebid AHI, Mobarez MA, Ramadan RA, Mahmoud MA. Impact of a Clinical Pharmacist Intervention Program on the Follow-up of Type-2 Diabetic Patients. Hosp Pharm [Internet]. 2022;57(1):76–82. Available from: http://ovidsp.ovid.com/ovidweb.cgi?T=JS&PAGE=reference&D=pmnm6&NEWS=N&AN=35521013
5. Chinn S. A simple method for converting an odds ratio to eeect size for use in meta-analysis. STATISTICS IN MEDICINE Statist Med [Internet]. 2000 [cited 2025 Aug 26];19:3127–31. Available from: https://onlinelibrary.wiley.com/terms-and-conditions
6. Cochrane Handbook for Systematic Reviews of Interventions | Cochrane Training [Internet]. [cited 2023 May 1]. Available from: https://training.cochrane.org/handbook/
7. McGuinness LA, Higgins JPT. Risk-of-bias VISualization (robvis): An R package and Shiny web app for visualizing risk-of-bias assessments. Res Synth Methods. 2021 Jan 1;12(1):55–61.
8. Chapter 14: Completing 'Summary of findings' tables and grading the certainty of the evidence | Cochrane [Internet]. [cited 2025 Aug 24]. Available from: https://www.cochrane.org/authors/handbooks-and-manuals/handbook/current/chapter-14
9. Stamm-Balderjahn S, Brunger M, Michel A, Bongarth C, Spyra K. The Efficacy of Goal Setting in Cardiac Rehabilitation-a Gender-Specific Randomized Controlled Trial. Dtsch Arztebl Int [Internet]. 2016;113(31–32):525–31. Available from: http://ovidsp.ovid.com/ovidweb.cgi?T=JS&PAGE=reference&D=med13&NEWS=N&AN=27581505
10. Sarycheva AA, Nebieridze D V, Kamyshova T V. Is it possible to improve the adherence to treatment of hypertension and dyslipidemia in patients without clinical manifestations of atherosclerosis? Rational pharmacotherapy in cardiology [Internet]. 2017;13(5):602-608-. Available from: https://www.cochranelibrary.com/central/doi/10.1002/central/CN-01622736/full
11. Buhse S, Kuniss N, Liethmann K, Muller UA, Lehmann T, Muhlhauser I. Informed shared decision-making programme for patients with type 2 diabetes in primary care: cluster randomised controlled trial. BMJ Open [Internet]. 2018;8(12):e024004-. Available from:

http://ovidsp.ovid.com/ovidweb.cgi?T=JS&PAGE=reference&D=med15&NEWS=N&AN=30552272

12. Lyons I, Barber N, Raynor DK, Wei L. The Medicines Advice Service Evaluation (MASE): a randomised controlled trial of a pharmacist-led telephone based intervention designed to improve medication adherence. BMJ Qual Saf [Internet]. 2016;25(10):759–69. Available from: http://ovidsp.ovid.com/ovidweb.cgi?T=JS&PAGE=reference&D=med13&NEWS=N&AN=26755665

13. Page MJ, McKenzie JE, Bossuyt PM, Boutron I, Hoffmann TC, Mulrow CD, et al. The PRISMA 2020 statement: an updated guideline for reporting systematic reviews. BMJ 2021;372:n71. doi: 10.1136/bmj.n71. This work is licensed under CC BY 4.0. To view a copy of this license, visit https://creativecommons.org/licenses/by/4.0/
